# Intraoperative Arterial Waveform Morphology Trajectories and Prediction of Postoperative Acute Kidney Injury: A Latent-Class Analysis of the VitalDB Registry

**DOI:** 10.3390/jcm15176871

**Published:** 2026-09-04

**Authors:** Mesher Ensarioğlu, Alperen Kutay Yıldırım

**Affiliations:** 1 Department of Anaesthesiology and Reanimation, Gülhane Training and Research Hospital, University of Health Sciences, 06010 Ankara, Türkiye; 2 Department of Cardiovascular Surgery, Gülhane Training and Research Hospital, University of Health Sciences, 06010 Ankara, Türkiye; drayildirim@yahoo.com

**Keywords:** acute kidney injury, arterial pressure waveform, intraoperative hypotension, clinical prediction model, dynamic arterial elastance, latent class analysis, VitalDB

## Abstract

**Background:** Intraoperative hypotension is an established risk factor for postoperative acute kidney injury (AKI), but whether arterial waveform morphology adds prognostic information beyond the hypotension burden is unknown. **Methods:** In this study, 2587 adult surgical cases from the Vital Signs Database (VitalDB) with continuous invasive arterial waveforms and creatinine-based (KDIGO) AKI ascertainment were analyzed retrospectively. Maximal systolic upstroke (dP/dtmax), a late-diastolic pressure-decay descriptor (τ), and a waveform-derived dynamic arterial elastance (Eadyn), modeled as latent-class trajectories and as case-level features, were added to a base logistic model of log-transformed hypotension burden (time-weighted MAP < 65 mmHg), age, sex, and baseline eGFR. **Results:** AKI occurred in 143 cases (5.5%); the base model had an AUC of 0.725 (95% CI 0.680–0.770). A five-class dP/dtmax solution yielded distinct phenotypes whose membership was nominally associated with AKI (likelihood-ratio *p* = 0.02) but did not improve discrimination (change in AUC (ΔAUC), 0.008, 95% CI −0.005 to 0.021; optimism-corrected 0.002), and the association was attenuated after clinical adjustment (*p* = 0.13); τ showed no stable trajectory structure. No feature improved discrimination: the optimism-corrected ΔAUC was ≤0.007, upper confidence limits did not exceed 0.03 on any adequately fitting reference model, and the signal disappeared after adjustment for procedure type and comorbidity. The likelihood-ratio test had 80% power for a ΔAUC of 0.009. **Conclusions:** Arterial waveform morphology did not improve prediction of postoperative AKI beyond the hypotension burden; overall, these data exclude an incremental gain larger than approximately 0.02–0.03 AUC units.

## 1. Introduction

Acute kidney injury (AKI) is among the most common postoperative complications, and postoperative increases in serum creatinine are linked to prolonged hospitalization, progression of kidney disease, and increased mortality [1]. Under the Kidney Disease: Improving Global Outcomes (KDIGO) framework, AKI is defined by an acute rise in serum creatinine or a decrease in urine output within a defined postoperative window. Its prevention has become a priority in perioperative management [2]. Among potentially modifiable intraoperative exposures, hypotension is the best characterized. In large surgical cohorts, both absolute thresholds (mean arterial pressure [MAP] below 65 mmHg) and relative reductions from baseline are associated with AKI [3,4]. Systematic reviews and international consensus statements indicate that mean arterial pressures below roughly 60–70 mmHg pose renal and myocardial risks that increase with duration [5,6]. Consequently, the burden of hypotension has become the reference against which new markers of renal risk are evaluated.

Open, high-resolution intraoperative registries have enabled investigations beyond mean blood pressure. The Vital Signs DataBase (VitalDB) provides beat-to-beat invasive arterial waveforms and perioperative outcomes for several thousand surgical patients. From these waveforms, a set of physiologically interpretable morphological descriptors can be derived [7]. The maximal systolic upstroke slope (dP/dtmax) reflects the rate of ventricular pressure development, although its peripheral (radial or femoral) version is load-dependent and tracks pulse pressure and arterial stiffness as much as contractility; the diastolic pressure-decay time constant (τ) is an empirical descriptor of the rate of late-diastolic pressure decay, conceptually related to the time constant of the arterial Windkessel; and dynamic arterial elastance (Eadyn), the ratio of pulse-pressure variation to stroke-volume variation, reflects ventriculo-arterial coupling and is used at the bedside to assess the arterial-pressure response to volume expansion [8,9,10,11,12]. dP/dtmax and Eadyn are displayed alongside the Hypotension Prediction Index, a machine-learning algorithm that predicts intraoperative hypotension from changes in the arterial waveform, as companion decision-support parameters on the same monitoring platform [13]. Because these features characterize how the circulation generates pressure rather than the pressure itself, they are potential markers of a patient’s hemodynamic state during surgery.

It remains unclear whether waveform morphology provides prognostic information about organ injury independently of the hypotension burden to which it may contribute. The arterial waveform encodes information about near-future pressure behavior, and thus machine-learning models built on intraoperative waveforms often predict hypotension with high accuracy [14]. Modeling the temporal pattern of MAP as latent-class trajectories, a group-based approach that resolves a heterogeneous set of longitudinal courses into a small number of representative patterns, has also been shown to improve prediction of major postoperative complications relative to conventional summaries [15,16]. Evidence also points the other way: machine-learning models restricted to demographic, laboratory, and summary blood-pressure features already predict post-surgical AKI, leaving open how much morphology-specific signal remains once these predictors are accounted for [17].

For this study, a retrospective analysis of the VitalDB registry was conducted to assess whether the intraoperative morphology of the invasive arterial waveform improves prediction of postoperative AKI beyond a model based on intraoperative hypotension burden and baseline characteristics. Morphology was represented in two ways: as latent-class temporal trajectories of dP/dtmax and τ over the course of surgery, and as case-level dynamic arterial elastance.

Additionally, sensitivity analyses of the reference model, the link between latent class and outcome, and the time axis on which trajectories are defined were to be reported. We distinguish throughout between prespecified and exploratory comparisons.

## 2. Materials and Methods

### 2.1. Study Design, Data Source, and Ethical Approval

A retrospective secondary analysis of the Vital Signs Database (VitalDB), an open, high-resolution registry of intraoperative physiological waveforms with linked perioperative clinical and laboratory data from 6388 surgical cases at a tertiary academic center, was conducted [7]. The registry was assembled with the approval of the Seoul National University Hospital Institutional Review Board (H-1408-101-605) and is fully de-identified and publicly available. The present study was evaluated by the Scientific Research Evaluation Board of Gülhane Training and Research Hospital, University of Health Sciences, on 14 July 2026 (evaluation number 12). Because the study was a computational secondary analysis of a de-identified, publicly available registry, the board waived the requirement for additional ethics approval and for informed consent, and the data were used in accordance with the registry’s data-use terms. As an observational prediction study, it is reported in accordance with the STROBE statement for observational studies [18]. The analysis plan submitted to the board specified the primary comparison (a reference logistic model of hypotension burden and clinical covariates versus the same model augmented with latent-class trajectory membership), the three morphology features, the KDIGO creatinine outcome with KDIGO stage as a secondary outcome, and the evaluation metrics (AUC, reclassification, and decision-curve analysis).

### 2.2. Participants

Cases were included if they contained a continuously recorded invasive arterial pressure waveform, if the patient was an adult (≥18 years) operated on under general anesthesia, and if they were evaluable for the AKI outcome, defined as having a baseline serum creatinine and at least one postoperative serum creatinine measured within 7 days of the end of surgery. Cases with pre-existing end-stage renal disease (a baseline serum creatinine ≥ 4.0 mg/dL or an estimated glomerular filtration rate (eGFR) < 15 mL/min/1.73 m^2^) were excluded, as the creatinine-based KDIGO criteria are uninformative in dialysis-dependent patients. Cases in which no physiologically valid arterial beats could be extracted or that failed the signal-quality thresholds defined in Section 2.6 were excluded from the analysis cohort. Throughout the paper, ‘derived cohort’ denotes the AKI-evaluable, non-ESRD cohort before waveform processing and ‘analysis cohort’ the subset with an evaluable waveform; all models were fitted in the analysis cohort.

To characterize selection, all adult waveform cases excluded at any step were compared with the analysis cohort on the registry covariates, separately for each reason for exclusion. Because the dominant reason for exclusion was the absence of a postoperative creatinine, the determinants of having a postoperative creatinine measured within 7 days were examined among adult, non-ESRD waveform cases with a baseline creatinine by logistic regression on age, sex, body mass index, ASA class, emergency status, eGFR, operative duration, intensive-care admission, and procedure category.

### 2.3. Outcome

The primary outcome was postoperative AKI, defined by the KDIGO clinical practice guideline serum-creatinine criterion [2], with the preoperative serum creatinine used as the baseline. AKI was defined as an acute rise in serum creatinine of ≥0.3 mg/dL within 48 h of the end of surgery, or an increase to ≥1.5 times baseline within 7 days.

Severity was staged as follows: stage 3, an acute rise with an increase to ≥3.0 times baseline or a peak creatinine ≥4.0 mg/dL; stage 2, an acute rise with an increase to ≥2.0 times baseline; and stage 1, an acute rise that did not meet the criteria for a higher stage. Renal-replacement-therapy and urine-output data are not available in the registry, so the KDIGO stage 3 criterion for dialysis initiation and the urine-output criteria at every stage could not be applied. Postoperative intensive-care admission and in-hospital death, which the registry records, are reported as indicators of clinical severity, and KDIGO stage ≥2 was analyzed as a secondary outcome. Baseline eGFR was computed from the baseline creatinine, age, and sex using the 2021 race-free CKD-EPI creatinine equation [19].

### 2.4. Hypotension Burden and Clinical Covariates

MAP was derived beat by beat from the invasive arterial waveform, and intraoperative hypotension burden was quantified as the time-weighted area under a MAP of 65 mmHg (TWA < 65, in mmHg·min), each beat weighted by its duration [3,6]. For sensitivity analyses, the per-minute MAP series was used to compute the time-weighted area and the number of minutes below 55, 60, 65, and 70 mmHg and the minimum MAP; the per-minute TWA < 65 agreed with the beat-level value (r = 0.98 on the logarithmic scale).

Clinical covariates were taken from the registry case table: age, sex, body mass index, ASA physical status, emergency status, hypertension, diabetes, procedure category (the registry’s 11 categories, with the two smallest merged into ‘others’), operative duration, crystalloid and colloid volumes, estimated blood loss, red-cell transfusion, and intraoperative bolus doses of ephedrine, phenylephrine, and epinephrine. Crystalloid volume was expressed per hour of surgery; colloid, transfusion, and each vasopressor were analyzed as any-versus-none indicators and blood loss on the logarithmic scale. Nephrotoxic co-medication, contrast exposure, and postoperative fluid management are not recorded in VitalDB and thus could not be utilized.

### 2.5. Waveform Processing and Morphology Features

**Figure 1:**
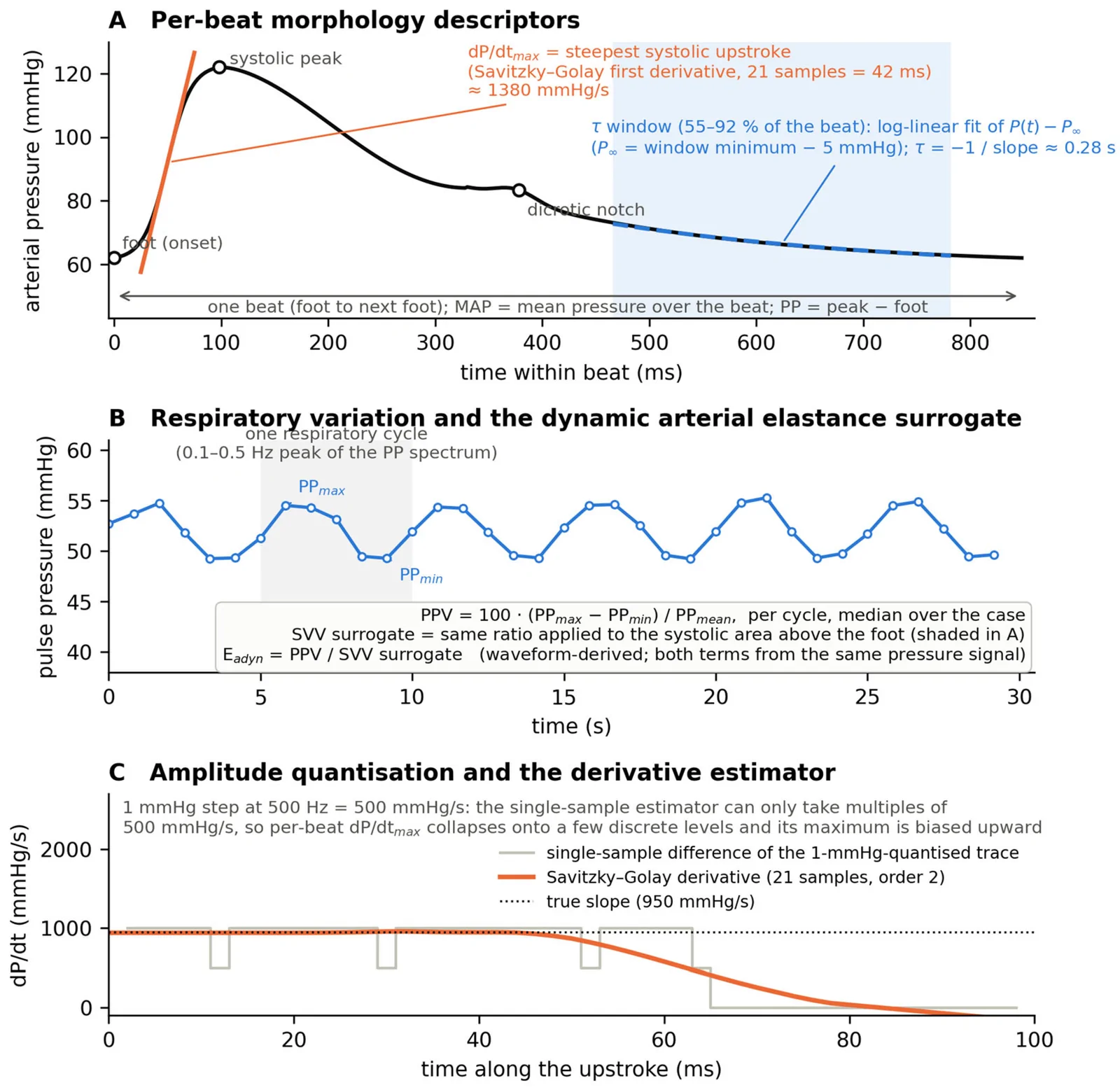
Waveform features and the quantization problem. (**A**) Per-beat descriptors: the foot, systolic peak, and dicrotic notch; dP/dtmax as the steepest systolic upstroke estimated by a Savitzky–Golay first derivative; and τ from a log-linear fit of the late-diastolic decay (55–92% of the beat). (**B**) Respiratory variation of pulse pressure (PP): pulse-pressure variation (PPV) and the systolic-area-based stroke-volume variation (SVV) are computed per respiratory cycle, and Eadyn = PPV/SVV. (**C**) A 1 mmHg amplitude step at 500 Hz equals 500 mmHg/s, so a single-sample difference of the quantized trace takes only multiples of 500 mmHg/s, whereas the Savitzky–Golay derivative recovers the continuous slope.

The SNUADC/ART waveform for each case was restricted to the intraoperative window (surgery start to end) and analyzed beat by beat at its native 500 Hz resolution (Figure 1). Physiologically valid signal segments were identified by rejecting flat-line, over-damped, and out-of-range stretches, and individual beats were detected within these segments. For each accepted beat, morphological descriptors were computed: dP/dtmax, τ, and Eadyn. τ was estimated per beat by a log-linear fit of the pressure decay over the mid-to-late diastolic window (55–92% of the beat), with the asymptote fixed 5 mmHg below the diastolic minimum. It is reported as an empirical late-diastolic decay descriptor rather than a lumped-parameter (Windkessel) time constant [10]. Eadyn was computed as the ratio of pulse-pressure variation to a stroke-volume-variation surrogate, the latter derived from beat-to-beat variation in systolic area rather than a calibrated stroke-volume signal, with the respiratory oscillation identified spectrally from the pulse-pressure signal (0.1–0.5 Hz). Its absolute value is therefore not directly comparable to device-derived Eadyn, and because both terms of the ratio are obtained from the same pressure signal, mathematical coupling between them cannot be excluded. Supporting quantities, including MAP, pulse pressure, heart rate, and beat-to-beat coefficients of variation, were also recorded.

A methodological correction was required for dP/dtmax because the SNUADC/ART channel has an amplitude resolution of approximately 1 mmHg. At 500 Hz, a 1 mmHg step corresponds to 500 mmHg/s, so estimating dP/dtmax with a single-sample finite difference collapses the estimate onto a small number of discrete levels. This was verified before extraction, as 200 linear upstrokes with true slopes drawn uniformly from 500–2200 mmHg/s were sampled at 500 Hz and rounded to 1 mmHg.

The single-sample estimator returned five distinct values (500, 1000, 1500, 2000, and 2500 mmHg/s), was biased upward (median 1500 mmHg/s), and produced values above the true maximum, whereas a Savitzky–Golay first derivative recovered 161 distinct values spanning the true range (500–2205 mmHg/s). To recover a continuous, physiologically interpretable derivative, dP/dtmax was therefore computed as the maximum of a Savitzky–Golay first derivative over the systolic upstroke (window length 21 samples, corresponding to 42 ms at 500 Hz; polynomial order 2) [20]. Values outside the physiological range of 30–5000 mmHg/s were then discarded as artifactual. Because the smoothing order attenuates the peak slope, a sensitivity re-extraction with a third-order polynomial over the same window was performed in the first 300 cohort cases. The absolute value of dP/dtmax is estimator-dependent, and it is analyzed as a relative index.

### 2.6. Signal Quality Control and Case-Level Summaries

Validated per-beat values were aggregated into 1 min bins to yield, for each case, a per-minute median time series for each feature throughout the duration of surgery. Signal quality control was then applied at the case level using fixed thresholds. A case was retained only if the fraction of usable (physiologically valid) signal was at least 0.50. Two additional conditions were screened: a damped or artifactual trace, operationalized as a case-median MAP below 45 mmHg together with a usable fraction below 0.80; and a τ estimate outside the plausible quality-control range of 0.12–1.50 s. Cases meeting the damped-trace condition or failing the usable-fraction threshold were excluded; cases flagged only for τ were retained. Case-level summaries were the per-case median and coefficient of variation of each beat-level feature together with PPV, the SVV surrogate, and Eadyn.

### 2.7. Latent-Class Trajectory Models

For each trajectory-eligible morphology feature (dP/dtmax and τ), the per-case one-minute series was placed on a normalized operative-time axis rescaled to the interval [0, 1] for each case’s duration and re-gridded into 20 equally spaced bins by taking the median within each bin, so that operations of differing length were represented on a common temporal frame. Latent-class linear mixed models were then fitted with the hlme function of the R version 4.5.3 using the lcmm package, version 2.2.2 [21]. dP/dtmax was modeled on its native scale and τ on the natural-log scale, each with a natural cubic spline of time (3 degrees of freedom) as class-specific fixed effect and a random intercept and slope. Models were fitted for one to five latent classes (G = 1–5, the prespecified range); for each G ≥ 2, the maximum-likelihood solution was obtained by a grid search over 30 random initializations with 30 iterations. The number of classes was selected as the solution with the lowest Bayesian information criterion among those that converged and in which every class contained at least 5% of cases; when no multi-class solution satisfied both constraints, the feature was reported as showing no stable multi-class trajectory structure. Classification quality of the selected solution was summarized by the relative entropy, the average posterior probability of assignment, and the odds of correct classification. Eadyn, defined as a single per-case summary of respiratory pressure variation rather than a continuous within-case series, could not be subjected to trajectory modeling.

Absolute-time sensitivity analyses were additionally performed. Rescaling operative time to the unit interval discards absolute duration and stretches or compresses segments unevenly across cases, so latent classes could in principle reflect operative length rather than within-case change. Two analyses that keep absolute time were therefore performed for dP/dtmax. First, the per-case series of 5 min medians (variable length, truncated at 480 min) were clustered by k-medoids (partitioning around medoids) on the dynamic-time-warping distance with a Sakoe–Chiba band of 60 min, for k = 2–6; the silhouette width and a minimum cluster size of 5% guided the choice of k, and the three- and four-cluster solutions were carried forward [22,23]. Second, a group-based trajectory model with the same natural-spline time basis was fitted on absolute minutes by the expectation–maximization algorithm (a finite mixture of spline regressions with class-specific residual variance; K = 1–5, class number chosen by the criteria above) [15]. Cluster and class membership were then tested as predictors in exactly the same way as the normalized-time classes.

### 2.8. Statistical Analysis

The reference model for postoperative AKI was a logistic regression of the logarithm-transformed hypotension burden, log(1 + TWA < 65), together with age, sex, and baseline eGFR; this base model was specified to represent hypotension exposure adjusted for the principal baseline determinants of AKI. Its functional form was examined with restricted cubic splines (3 and 4 knots at the default quantiles) for log-burden and for age; with alternative hypotension metrics (time-weighted area or minutes below 55, 60, 65, and 70 mmHg, minimum MAP, and TWA < 55 added to TWA < 65); with baseline creatinine in place of eGFR and with an age-residualized eGFR; with an age × burden interaction; and with an expanded clinical model that added emergency status, ASA class ≥ 3, body mass index, hypertension, diabetes, log operative duration, crystalloid rate, colloid use, log blood loss, red-cell transfusion, each vasopressor, and procedure category [24]. Crystalloid volume (missing in 2.5%) was median-imputed, blood loss (missing in 24%) was median-imputed with a missingness indicator, and all other variables were complete, so that all analyses were otherwise complete-case.

The prespecified primary comparison was the base model versus the base model plus dP/dtmax latent-class membership; the τ trajectory and Eadyn were prespecified secondary features, and the remaining case-level summaries (medians and coefficients of variation of dP/dtmax, τ, MAP and pulse pressure, PPV, and the SVV surrogate) were exploratory. Each representation was added to the base model in turn. The contribution of the added terms was tested by the likelihood-ratio (LR) test [25,26]. Discrimination was quantified by the area under the receiver-operating-characteristic curve (AUC), and the change in AUC (ΔAUC) with its DeLong confidence interval and test is reported for comparability [27]. Incremental reclassification was quantified by the integrated discrimination improvement (IDI) with bootstrap confidence intervals (1000 replications) [28]. Multiplicity across the morphology family (ten LR tests) was controlled by the Benjamini–Hochberg false-discovery rate [29]. As modal class assignment ignores classification uncertainty and biases associations toward the null, the class–outcome association was re-estimated in three bias-adjusted ways: with the posterior class probabilities as covariates, with 200 pseudo-class draws from the posterior combined by Rubin’s rules, and with the Bolck–Croon–Hagenaars (BCH) correction, in which each case enters the outcome model once per class with weights given by the inverse of the estimated classification-error matrix and cluster-robust standard errors [30,31,32,33].

Due to ΔAUC, IDI, and LR statistics being computed on the data used to fit the models, each base and extended model was internally validated by Harrell’s bootstrap (1000 resamples), and the AUC, ΔAUC, IDI, and calibration slope were corrected for optimism [34,35]. Calibration-in-the-large, the calibration slope, the Brier score, and the maximum deviation between observed and predicted risk are reported for the models, and decision-curve analysis compared their net benefit across threshold probabilities [36]. All incremental analyses were also repeated on the alternative base models. All tests were two-sided, with significance set at 0.05. Continuous variables were summarized as median (interquartile range).

### 2.9. Sample Size and Precision

The study used a fixed registry, so no a priori sample-size calculation was possible, and adequacy was judged from the number of events. With 143 events and four parameters, the base model had 36 events per variable, which satisfies conventional criteria for estimating a model of this size yet does not establish the precision of the incremental comparisons [37].

The minimum detectable increment was estimated by simulation at the actual sample size, with outcomes being regenerated from the fitted base linear predictor plus a synthetic standard-normal marker of increasing strength. The base and extended models were then refitted, and the power of the LR test and of the DeLong test was estimated from 300 replications. The ΔAUC and IDI at which power reached 80% and 90% are reported.

### 2.10. Software and Code

Feature extraction and signal processing were performed in Python 3.14.3; individual beats were detected with the peak-finding routine of the SciPy signal-processing library (SciPy 1.17.1), and subsequent processing used NumPy 2.4.3, pandas 2.3.3, and the VitalDB 1.7.2 interface [38,39]. Trajectory modeling was conducted in R with the lcmm package, and the outcome models in R with pROC [21,40,41]. The sensitivity analyses described above (splines, three-step linkage, dynamic-time-warping clustering, the group-based trajectory model, bootstrap validation, decision curves, and the power simulation) were implemented in Python with NumPy and SciPy. Figures were generated with Matplotlib 3.10.8 [42].

## 3. Results

### 3.1. Derivation of the Cohort and Comparison with Excluded Cases

**Figure 2:**
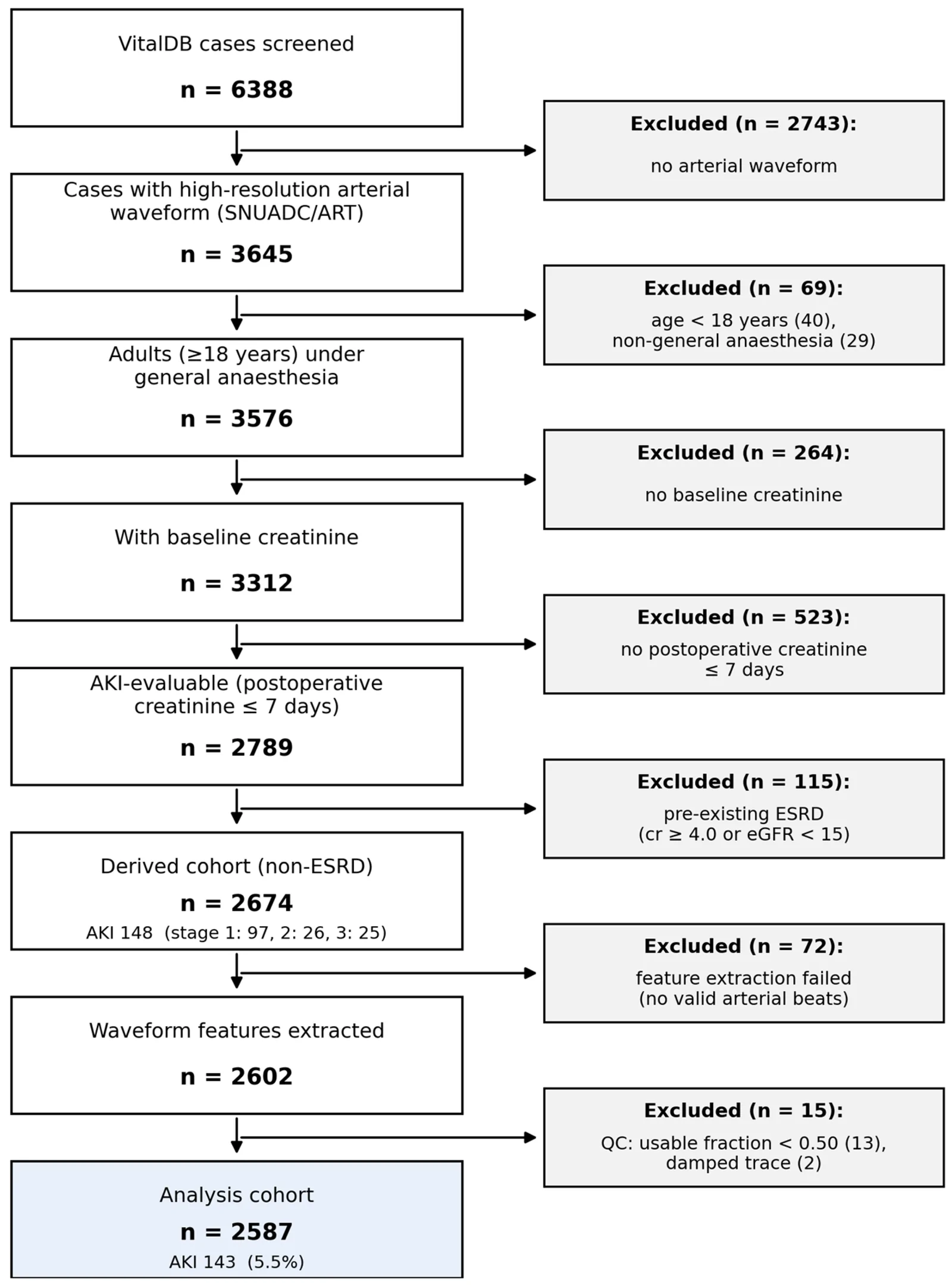
Study flow chart. AKI: acute kidney injury; cr: serum creatinine; ESRD: end-stage renal disease; eGFR: estimated glomerular filtration rate; SNUADC/ART: invasive arterial pressure channel; QC: signal quality control.

Of the 6388 surgical cases present in the registry, 3645 contained a high-resolution SNUADC/ART arterial waveform; 69 were excluded because patients were younger than 18 years (n = 40) or the operation was not performed under general anesthesia (n = 29). Requiring a baseline preoperative creatinine removed a further 264 cases, and requiring at least one postoperative creatinine within 7 days of the end of surgery removed 523 more, leaving 2789 AKI-evaluable cases; excluding 115 cases with pre-existing end-stage renal disease (baseline creatinine ≥ 4.0 mg/dL or eGFR < 15 mL/min/1.73 m^2^) yielded a derived cohort of 2674 cases, in which postoperative AKI occurred in 148 (5.5%; KDIGO stage 1, 97; stage 2, 26; stage 3, 25). Waveform feature extraction failed in 72 cases in which no physiologically valid arterial beats could be recovered, and a further 15 cases were removed at signal quality control (13 for a usable signal fraction below 0.50 and 2 as damped-trace suspects); 9 cases flagged only for an out-of-range τ were retained. The final analysis cohort comprised 2587 cases with 143 AKI events (5.5%; stage 1, 93; stage 2, 25; stage 3, 25) (Figure 2).

Patients without a postoperative creatinine within 7 days (n = 523) were younger (median 56 vs. 62 years), more often female (61% vs. 41%), had shorter operations (median 90 vs. 160 min), received less crystalloid, were less often admitted to intensive care (12% vs. 34%), and more often underwent procedures in the heterogeneous ‘other’ category; of the 511 non-ESRD cases among them, 451 (88%) did not have a postoperative creatinine. Among adult non-ESRD waveform cases with a baseline creatinine, a postoperative creatinine was measured within 7 days in 84.0% and was associated with operative duration (odds ratio 2.44 per log-minute, 95% CI 2.05–2.90), intensive-care admission (2.85, 2.05–3.96), male sex (1.74, 1.40–2.17), age (1.02 per year, 1.01–1.03), and procedure category (model AUC 0.79), but not with ASA class, emergency status, or baseline eGFR. The 87 derived-cohort cases lost at feature extraction or quality control had the same AKI rate as analyzed cases (5.7% vs. 5.5%, *p* = 0.81) and similar age, eGFR, ASA distribution, and emergency proportion, but shorter operations (median 103 vs. 160 min, *p* < 0.001); the 15 cases removed at quality control had a low usable signal fraction (median 0.41) and, as expected, hypotension burden could not be computed for cases excluded before waveform processing (Supplementary Table S1).

### 3.2. Cohort Characteristics

The cohort was predominantly of normal baseline renal function (median eGFR 95.2 mL/min/1.73 m^2^, interquartile range 83.6–104.0). Signal quality in the analysis cohort was high, with a median usable signal fraction of 0.98 (interquartile range 0.96–0.99) and a median of 11,018 (7082–16,739) analyzed beats per case over a median analyzed duration of 160 (105–240) min. Case-level medians of the derived features were within physiologically plausible limits: heart rate 72 (63–81) bpm, mean arterial pressure 83 (76–90) mmHg, pulse pressure 54 (48–61) mmHg, dP/dtmax 737 (621–884) mmHg/s, τ 0.24 (0.21–0.28) s, and dynamic arterial elastance 0.28 (0.21–0.40). The intraoperative hypotension burden had a right-skewed distribution: the time-weighted area under a MAP of 65 mmHg was 10.6 (0.4–67.7) mmHg·min, and the minimum MAP was 52 (45–60) mmHg. A sensitivity re-extraction of dP/dtmax with a third-order Savitzky–Golay derivative raised the cohort median by about 7% (from 737 to approximately 790 mmHg/s), confirming that the low absolute value is not an artifact of the smoothing order (Table 1). Vasopressor boluses were given in 62.8% of cases, colloid in 11.2%, and red cells in 9.1%; 33.6% of patients were admitted to intensive care, and 0.7% died in hospital. Patients who developed AKI were more often admitted to intensive care (61.5% vs. 31.9%) and died more often (2.1% vs. 0.6%) than those who did not, and intensive-care admission rose from 52% in stage 1 to 80% in stages 2 and 3.

### 3.3. Latent-Class Trajectories of dP/dtmax and τ

Latent-class mixed models of the dP/dtmax trajectory over normalized operative time converged for all one-to-five-class solutions. The Bayesian information criterion decreased monotonically with the number of classes, and the smallest-class share remained above the 5% floor at every solution (6.5% at five classes); the five-class solution, the largest in the prespecified range, was therefore selected as the lowest-BIC model that satisfied the minimum-class and convergence constraints. The sample-size-adjusted BIC and the integrated-completed-likelihood BIC also decreased monotonically across one-to-five classes, in parallel with the BIC, thus no information criterion could identify an optimal class number. The class number therefore is set by the prespecified range and the minimum-class constraint rather than by fit. For the selected five-class solution, classification quality was adequate: average posterior probabilities ranged from 0.85 to 0.92, odds of correct classification ranged from 6.1 to 94, and relative entropy was 0.846 (Table 2).

**Figure 3:**
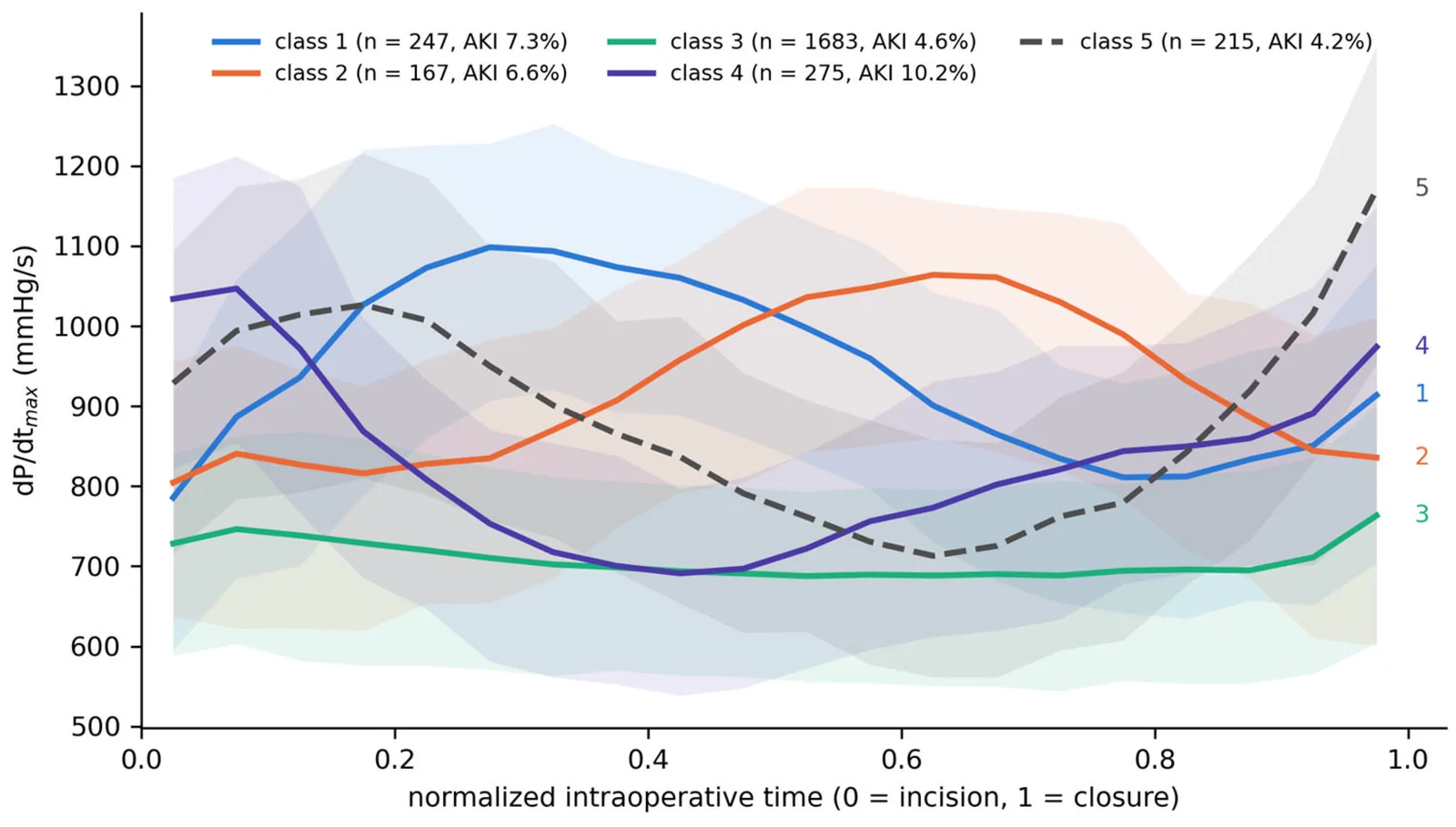
Class-mean intraoperative dP/dtmax trajectories (five-class solution) over normalized operative time (0 = incision, 1 = closure). Lines are class-mean trajectories; shaded bands are the interquartile range (n = 2587).

The five phenotypes were physiologically distinct (Table 3, Figure 3). Class 1 (247 cases, 9.5% of the cohort) rose from 786 mmHg/s to an early peak of about 1100 mmHg/s within the first third of surgery, declined to about 810 mmHg/s, and recovered to 914 mmHg/s by closure; class 2 (167 cases, 6.5%) rose to a mid-to-late peak (805 → 1064 → 836 mmHg/s); class 3, the dominant phenotype (1683 cases, 65%), followed a persistently low-to-moderate, essentially flat course (728 → 688 → 763 mmHg/s); class 4 (275 cases, 10.6%) began at a high level, fell to the deepest mid-surgery trough of any class, and recovered toward closure (1034 → 691 → 974 mmHg/s); and class 5 (215 cases, 8.3%) declined to a late-surgery trough and then rose steeply at closure (929 → 713 → 1170 mmHg/s). AKI rates were 7.3%, 6.6%, 4.6%, 10.2%, and 4.2% in classes 1–5, respectively (χ^2^ = 16.9, 4 df, *p* = 0.002). Class 4, the class with the highest AKI rate, also had the highest hypotension burden (median TWA < 65 21.8 mmHg·min vs. 9.2–11.4 mmHg·min in the other classes; Kruskal–Wallis *p* = 0.013), the lowest minimum MAP, the longest operations (median 185 min), and the highest proportions of ASA class ≥ 3 (20%), red-cell transfusion (16%), and vasopressor use (71%). With 9–28 events per class outside the dominant class, the class-specific rates are considered to be imprecise.

Latent-class models of the log-transformed τ trajectory converged for all solutions but did not yield a stable multi-class structure: at every solution with two or more classes, the smallest class fell below the 5% floor (ranging from 3.1% at two classes to 1.6% at five classes), so no multi-class solution satisfied both constraints. τ was therefore reported as showing no stable multi-class trajectory structure and was not carried forward as a trajectory predictor.

### 3.4. Base Model

In the base logistic model for postoperative AKI (log-transformed hypotension burden, age, sex, and baseline eGFR), a higher hypotension burden (odds ratio 1.439, 95% CI 1.324–1.563; *p* < 0.001) and male sex (2.022, 1.363–3.001; *p* < 0.001) were associated with AKI; older age was associated with slightly lower odds (0.979 per year, 0.966–0.993; *p* = 0.003), and baseline eGFR was not independently associated (0.991 per mL/min/1.73 m^2^, 0.983–1.000; *p* = 0.061). The base model discriminated with an AUC of 0.725 (0.680–0.770). Optimism was small on internal validation: the optimism-corrected AUC was 0.718, the calibration slope was 0.97, calibration-in-the-large was exact, the largest deviation between observed and predicted risk across deciles of predicted risk was 0.021, and the Brier score was 0.049. With 143 events and four estimated parameters, the base model had 36 events per variable (Table 4).

AKI was most frequent after transplantation (24.2%, median age 47 years) and ‘other’ procedures (10.4%) and least frequent after gastric (0.8%) and minor resections (1.5%); crude age was unrelated to AKI (odds ratio 0.992 per year, 0.981–1.004), and after adjustment for procedure category, the age coefficient was null (0.997, 0.982–1.012; *p* = 0.66). Baseline eGFR is computed from age and creatinine and was collinear with age (r = −0.57); when baseline creatinine replaced eGFR, it was positively associated with AKI (1.69 per mg/dL, 1.18–2.42; *p* = 0.004) with identical discrimination (AUC 0.725), an age-residualized eGFR behaved like eGFR, and there was no age × burden interaction (*p* = 0.46).

### 3.5. Incremental Value of Waveform Morphology

Adding the dP/dtmax trajectory class to the base model did not improve prediction, although class membership was nominally associated with AKI. The likelihood-ratio test for the four added terms was significant (χ^2^ = 11.6, 4 df, *p* = 0.020; q = 0.051 after false-discovery-rate control), which reflects a lower adjusted risk in the dominant flat class than in the four non-flat classes (odds ratios relative to class 1: class 2, 0.92, 0.41–2.05; class 3, 0.59, 0.34–1.02; class 4, 1.24, 0.65–2.36; class 5, 0.56, 0.24–1.31), yet discrimination did not improve (ΔAUC 0.008, 95% CI −0.005 to 0.021; DeLong *p* = 0.24; optimism-corrected ΔAUC 0.002), and the IDI was 0.009 (95% CI 0.003–0.015; optimism-corrected 0.006). The calibration slope of the extended model was 0.94 after correction, and net benefit differed from the base model by at most 0.004 across thresholds of 2–30%. On the expanded clinical reference model, the association was attenuated (ΔAUC 0.001, −0.006 to 0.008; LR *p* = 0.13). The unselected five-class τ solution also did not contribute (ΔAUC −0.002; LR *p* = 0.83).

As dynamic arterial elastance is a single per-case summary rather than a within-case time series, it and the remaining case-level morphology summaries were evaluated by adding each, in turn, to the base model (Table 5). Eadyn, the prespecified secondary feature, was not predictive as a passive marker (odds ratio 2.51 per unit; *p* = 0.063; ΔAUC 0.005, 95% CI −0.004 to 0.013; LR *p* = 0.070). Among the exploratory summaries, three had likelihood-ratio tests that remained significant after false-discovery-rate control (median dP/dtmax, q = 0.041; coefficient of variation of pulse pressure, q = 0.032; coefficient of variation of dP/dtmax, q = 0.041), and three did not (pulse-pressure variation, median τ, and the coefficient of variation of MAP, q = 0.080–0.085). None of these statistically detectable associations translated into better prediction: the apparent ΔAUC was at most 0.008, the upper 95% confidence limit at most 0.018, and the optimism-corrected ΔAUC at most 0.007. For these results, no DeLong test approached significance, and the IDIs were 0.007 or less. Calibration slopes of the extended models were 0.94–0.97 after optimism correction, and their decision curves were similar to that of the base model, as the net benefit differed from the base model by at most 0.0045 (fewer than five net true positives per 1000 patients) for any representation, including the trajectory class (Supplementary Figure S1 and Supplementary Table S5).

### 3.6. Robustness to the Specification of the Reference Model

Nonlinearity of the burden term was assessed with restricted cubic splines: a four-knot spline for log-burden improved fit over the log-linear term (LR χ^2^ = 11.7, 2 df, *p* = 0.003; AIC 1003.7 vs. 1011.4; AUC 0.730), describing a relation that is flat at low burden and steeper at high burden (Supplementary Figure S3). A square-root transform performed similarly (AIC 1006.6; AUC 0.732), while the untransformed burden fitted poorly (AUC 0.699). Nonlinearity in age was limited (*p* = 0.037) and did not alter the other coefficients. Among single hypotension metrics, the beat-level TWA < 65 had the best fit (AIC 1011.4); thresholds of 55 mmHg (AUC 0.704 for the time-weighted area, 0.706 for minutes) and the minimum MAP (AUC 0.704) discriminated less well, but TWA < 55 carried some information beyond TWA < 65 when both were entered (odds ratio 1.21 per log unit, *p* = 0.009; AUC 0.727). Colloid administration was associated with AKI (odds ratio 2.70, 1.77–4.13), whereas the crystalloid rate (1.42 per L/h, *p* = 0.09) and any vasopressor bolus (0.70, *p* = 0.11) were not (AUC 0.752 with these terms). The expanded clinical model discriminated substantially better than the base model (AUC 0.837, 0.804–0.870; LR χ^2^ = 140, 22 df), with ASA class ≥ 3 (odds ratio 2.92), hypertension (1.68), red-cell transfusion (1.92), male sex (1.84), and procedure category as independent predictors, while the age coefficient was null (Supplementary Table S2).

None of the reference models with added morphology representation provided additional discrimination. Across the eleven specifications that fit at least as well as the base model, the largest ΔAUC for any prespecified or exploratory feature was 0.013, and the largest upper confidence limit was 0.028. For the expanded clinical model, the largest ΔAUC was 0.005 (upper limit 0.012), and the smallest LR *p*-value was 0.023 (pulse-pressure variation); the trajectory class (*p* = 0.13), median-dP/dtmax (*p* = 0.077), and associations with τ and coefficient of variation were no longer significant. Morphology features were essentially unrelated to intraoperative treatment, as their Spearman correlations with the crystalloid rate were 0.19 or less in absolute value, and vasopressor use (58–65%) and crystalloid rate (0.43–0.48 L/h) were similar across trajectory classes (Table 6; the complete set of increments for every specification is given in Supplementary Table S8).

### 3.7. Robustness of the Class–Outcome Linkage and of the Time Axis

The result for the dP/dtmax trajectory class did not depend on modal assignment. The estimated classification-error matrix had diagonal elements of 0.78–0.95. With the posterior class probabilities as covariates, the LR test gave *p* = 0.026 (ΔAUC 0.008); across 200 pseudo-class draws, the pooled odds ratios were 0.99, 0.66, 1.18, and 0.55 for classes 2–5, respectively, relative to class 1 (all *p* > 0.15; mean LR χ^2^ 8.6, *p* = 0.07; mean ΔAUC 0.007); and the BCH-corrected estimates were 0.92, 0.52, 1.30, and 0.49 (Wald χ^2^ = 12.5, 4 df, *p* = 0.014). (Supplementary Table S3).

Absolute-time representations separated cases by the level rather than the shape of dP/dtmax. Dynamic-time-warping k-medoids favored two clusters by silhouette width (0.36; 0.26 for three), and the three-cluster solution comprised a low (median dP/dtmax 539 mmHg/s), a middle (740 mmHg/s), and a high-level cluster (1014 mmHg/s) with AKI rates of 6.4%, 4.3%, and 7.4%, respectively. Overall, cluster membership contributed little to the base model (ΔAUC 0.006, 95% CI −0.004 to 0.017; LR *p* = 0.037; optimism-corrected ΔAUC 0.003).

The group-based trajectory model on absolute time identified five nearly parallel classes (class means from about 480 to 1380 mmHg/s) (Supplementary Figure S2). The smallest class (138 cases, 5.3%) had a persistently high dP/dtmax (about 1250–1380 mmHg/s) and a wide pulse pressure (median 75 mmHg), and its members were older (median 71 years), more often ASA class ≥ 3 (28%), operated on as emergencies (23%), and transfused (15%). These patients had an AKI rate of 11.6%, and their class carried the only nominally significant trajectory signal in the study (ΔAUC 0.012, 95% CI −0.003 to 0.028; LR χ^2^ = 13.4, 4 df, *p* = 0.009; IDI 0.010; optimism-corrected ΔAUC 0.006). However, the signal disappeared once the expanded clinical model served as the reference (ΔAUC 0.002, −0.004 to 0.008; LR *p* = 0.26; dynamic-time-warping clusters: ΔAUC 0.001, *p* = 0.13) (Supplementary Table S4). Agreement between normalized-time and absolute-time classes was also weak, as the high-level absolute-time class recruited members from all five normalized-time classes.

### 3.8. Severity and Precision

With KDIGO stage ≥2 as the outcome (50 events), the base model discriminated with an AUC of 0.755 (0.680–0.830). The dP/dtmax trajectory class did not add discrimination (ΔAUC 0.012, −0.013 to 0.036; LR *p* = 0.064). Median dP/dtmax had a significant LR test (*p* = 0.001; IDI 0.020), but its ΔAUC of 0.012 had a wide confidence interval (−0.017 to 0.040), and the coefficient of variation of pulse pressure was borderline (ΔAUC 0.016, −0.001 to 0.032; LR *p* = 0.051) (Supplementary Table S7). Regarding precision, the observed standard errors of ΔAUC across the extended models were 0.003–0.008. In the simulation at the actual sample size, the LR test had 80% power to detect a marker producing a ΔAUC of 0.009 (IDI 0.006) and 90% power at a ΔAUC of 0.011, whereas the DeLong test reached 80% power only at a ΔAUC of 0.028 (Supplementary Table S6). The study was therefore considered adequately powered, with the primary LR test, to detect increments far smaller than those usually regarded as clinically meaningful, and the observed upper confidence limits (≤0.021 for the prespecified and exploratory features on the base model, ≤0.03 for any representation on any adequately fitting reference model) bound the incremental value with which the data are compatible.

## 4. Discussion

In this analysis of adult surgical cases from the VitalDB registry, intraoperative arterial waveform morphology did not improve prediction of postoperative acute kidney injury (AKI) beyond a model that included intraoperative hypotension burden, age, sex, and baseline renal function. This was observed for the dP/dtmax trajectory classes and for every case-level summary, including Eadyn. τ yielded no stable trajectory structure and was therefore assessed only as a case-level median. Adding any of these to the base model changed discrimination negligibly (apparent ΔAUC ≤ 0.008 and optimism-corrected ΔAUC ≤ 0.007 for every prespecified and exploratory representation). The cohort contained 143 AKI events, and the base model discriminated moderately well (AUC 0.725). The null is bounded rather than merely non-significant. The likelihood-ratio test, which is the appropriate test of incremental value in nested models, had 80% power to detect a ΔAUC of about 0.009. For the primary comparison, it reached nominal significance (*p* = 0.02; q = 0.05), because the dominant flat trajectory class carried a lower adjusted risk than the four non-flat classes, yet the gain in discrimination was 0.008 (upper confidence limit 0.021) and 0.002 after optimism correction, net benefit was unchanged, the three bias-adjusted three-step estimators gave the same answer, and the association was attenuated to *p* = 0.13 once procedure type, ASA class, emergency status, and transfusion entered the reference model.

The upper 95% confidence limits for ΔAUC did not exceed 0.021 on the base model or 0.03 on any better-fitting alternative reference model, and the one absolute-time signal, a small high-dP/dtmax-level class, likewise disappeared with clinical adjustment. These data therefore exclude an incremental gain larger than approximately 0.02–0.03 AUC units from the waveform-derived surrogates studied, and the decision curves show no net benefit from the smaller increments observed, without excluding a smaller gain.

The use of hypotension burden as the reference for predictive assessment is consistent with a large body of evidence linking intraoperative hypotension to AKI. In 33,330 non-cardiac operations, Walsh and colleagues identified a mean arterial pressure (MAP) threshold of 55 mmHg, below which AKI risk increased with cumulative exposure time [4]. Salmasi and colleagues showed that both absolute (MAP < 65 mmHg) and relative thresholds are associated with AKI and myocardial injury [3]. The base model presented here supports these results, as a time-weighted burden of MAP below 65 mmHg reproduced the expected association, providing internal confirmation that the pressure signal was captured adequately. Two refinements of the base model deserve further comments and explanation. The dose–response relation was convex on the logarithmic scale, flat at small burdens and steep at large ones, and exposure below 55 mmHg carried information beyond the 65 mmHg area, both consistent with the threshold-like behavior described by Walsh and colleagues; and the expanded clinical model, in which procedure category, ASA class, hypertension, and transfusion were strong predictors, discriminated far better (AUC 0.84) than hypotension burden and demographics alone. Neither refinement supported the role of morphology, with increments decreasing as the reference model improved.

Ren and colleagues applied group-based trajectory modeling to intraoperative MAP in a study of 789 patients undergoing major abdominal, urologic, or gynecologic surgery [16]. The study identified three hypotension trajectories distinguished by duration and recurrence and reported a graded exposure–response relationship with a composite outcome of AKI, delirium, unplanned ICU admission, and 30-day mortality. A key difference from our study was that Ren and colleagues modeled the temporal pattern of MAP, whereas we assessed the shape of the waveform. Both studies point to the achieved pressure and its temporal burden as determinants of renal risk.

Two additional VitalDB studies bear on this result. Lee and colleagues developed a machine-learning model for post-non-cardiac-surgery AKI and, on external validation in the VitalDB dataset, achieved an AUC of 0.757 using demographic, preoperative-laboratory, and summary intraoperative-blood-pressure features. This result is close to our 0.725 and reinforces that AKI in this population is largely predictable from summary hemodynamics and baseline characteristics, leaving little residual variance for waveform morphology to explain [17]. Shim and colleagues showed that biosignal waveforms and clinical data from the same VitalDB cohort predict impending intraoperative hypotension with high accuracy (AUC 0.94) [14]. Waveform morphology therefore carries information about the near-future behavior of the pressure itself, but predicting the next hypotensive event is mechanistically distinct from independently predicting a downstream organ injury. Our findings indicate that once the hypotension that actually occurred is quantified, the accompanying waveform offers no further information.

Dynamic arterial elastance shows the same pattern at the level of a single feature. Eadyn, the ratio of pulse-pressure variation to stroke-volume variation, has a well-established physiological rationale: Monge García and colleagues showed that it predicts the arterial-pressure response to volume expansion in preload-dependent patients, and it is used to guide the choice between fluids and vasopressors during hemodynamic resuscitation [12]. Its performance, however, is strongly context-dependent: in a meta-analysis, the pooled area under the summary ROC curve was 0.92 (95% CI 0.89–0.94) overall, 0.96 (0.94–0.98) in intensive-care patients, and 0.72 (0.67–0.75) in patients in the operating room, with reported thresholds ranging from 0.65 to 0.89 [43]. Eadyn was developed to guide a real-time therapeutic decision, which does not imply that it should behave as a passive marker of downstream organ injury. In our data, the waveform-derived surrogate, which is not the device-derived index validated in those studies, was not an independent predictor of AKI (adjusted OR 2.51, *p* = 0.063; ΔAUC 0.005). The morphological features we studied index how pressure is generated, whereas AKI is driven by the perfusion pressure actually achieved and the deficits therein, which the burden term already captures. Consistent with this, the trajectory phenotype that carried the highest AKI rate was not a distinct morphological signature but the class with the deepest mid-surgery fall in dP/dtmax, which also had the highest hypotension burden, the lowest minimum MAP, the longest operations, and the most transfusion and vasopressor use; it is best read as a marker of intraoperative instability that the burden term and the clinical covariates already capture, and thus no claim could be made regarding possible disassociation between morphology and burden.

The two trajectory signals that reached nominal significance are instructive, as both behaved as proxies. In normalized time, the residual association of class membership with AKI after adjustment for log-burden was concentrated in the unstable high-burden class described above and attenuated once procedural acuity entered the model. When absolute time was retained, both clustering approaches separated cases by the level of dP/dtmax rather than by its shape, and the small class with a persistently high dP/dtmax had a higher AKI rate. Peripheral dP/dtmax is not a load-independent index of contractility: in critically ill patients, it correlates at r > 0.9 with pulse pressure and total arterial stiffness and rises with volume loading in preload-dependent patients [8,9]. In our cohort, too, dP/dtmax correlated strongly with pulse pressure (r = 0.80), and the high-dP/dtmax class was older, had a wider pulse pressure, and was more often of high ASA class, operated on as an emergency, and transfused. Its association with AKI vanished when these characteristics entered the reference model, and the same attenuation affected the exploratory case-level dP/dtmax and pulse-pressure signals. This is precisely the mechanism by which a morphological feature would appear to add value in a study, by acting as a proxy for arterial stiffness, age, and clinical acuity.

The apparently paradoxical inverse association between age and AKI in the base model has the same explanation. AKI was concentrated in transplantation (24% of cases, median age 47 years) and in the heterogeneous ‘other’ category, in keeping with the high incidence of AKI after liver transplantation, while older patients predominated in gastric and colorectal surgery with AKI rates below 5% [44]. Crude age was unrelated to AKI, and the adjusted age coefficient was null once procedure category was included. The non-significance of eGFR likely reflects its near-collinearity with age in a cohort with a narrow range of renal functions. Baseline creatinine was associated with AKI when it replaced eGFR, and the incremental results were identical under either specification.

A further concern is that intraoperative treatment could confound a relation between waveform morphology and AKI: an early warning of hypotension is followed by fluid or vasopressor administration, and individualized pressure management reduces postoperative organ dysfunction, whereas restrictive fluid administration increases AKI [45,46]. The registry records the volumes of crystalloid and colloid, blood loss, transfusion, and vasopressor boluses, and these were included in the reference models. Colloid administration was associated with AKI in the fluids model and red-cell transfusion in the fully adjusted model, most plausibly as markers of bleeding and hemodynamic instability rather than as causes, but the morphology features were essentially uncorrelated with the amount of fluid or with vasopressor use, and the conclusion was unchanged whether or not treatment variables were in the model. VitalDB does not record nephrotoxic co-medication or postoperative fluid management, so these remain unmeasured.

One methodological observation applies more broadly to waveform-morphology studies of archived physiological data. The arterial channel in this registry is stored with approximately 1 mmHg amplitude resolution, and a dP/dtmax computed as a single-sample first difference is corrupted by this quantization: at a 500 Hz sampling rate, a 1 mmHg step corresponds to exactly 500 mmHg/s, so the estimator can only take integer multiples of that value. Quantization of this kind imposes artificial structure on derivative-based features, and a downstream model may mistake that structure for signal. We therefore computed dP/dtmax with the Savitzky–Golay estimator and recommend that studies mining archived waveforms report the source-channel amplitude resolution and the derivative estimator used.

This study has several limitations. AKI was ascertained using the KDIGO serum-creatinine criterion without urine-output or renal-replacement data, which may under-ascertain some events. However, this is standard for registry analyses and is unlikely to differentially bias a morphological signal; the secondary analysis of stage ≥2 AKI, and the gradient of intensive-care admission across stages, indicate that the creatinine-defined events were clinically relevant. The data derive from a single institution, limiting generalizability. Selection is a real concern: because a postoperative creatinine was measured mainly after longer, higher-acuity operations, the analysis cohort is enriched for such operations, and the results apply to the population in which renal surveillance is practiced rather than to all patients with an arterial line. Within that population, ascertainment was near-complete; the 87 cases lost at the waveform stage had the same AKI rate as analyzed cases, and hypotension burden could not be computed for cases excluded before waveform processing, so residual selection on burden cannot be excluded. The cohort was predominantly renally healthy and weighted toward elective surgery, with a modest AKI incidence. Thus, our results do not exclude a role for waveform morphology in higher-risk settings such as cardiac surgery, where reported incidences of AKI range from about 5% to over 40% depending on the definition, or in chronic kidney disease populations, where hemodynamic changes are more severe and renal reserve is lower [47,48]. The absence of a stable τ trajectory means that the diastolic-decay question is not fully resolved in this cohort.

Methodological limitations are also present. dP/dtmax is a relative, estimator-dependent index rather than an absolute measure of contractility. Values obtained from a peripheral fluid-filled line are sensitive to catheter and tubing damping and arterial compliance, and the median reported here is lower than published radial values. That difference is not attributable to the smoothing order and more likely reflects damping of the recording system together with anesthetic depression of contractility. A similar statement applies to τ and Eadyn, as τ is reported as an empirical late-diastolic decay descriptor rather than a lumped-parameter time constant, and Eadyn was derived from the arterial waveform as a provisional surrogate. This has a direct consequence for the scope of the negative result. Measurement error in a surrogate attenuates its apparent incremental value toward the null, so the study can rule out, with the precision quantified above, an incremental signal from these archived-data surrogates, but not from device-derived dP/dtmax and Eadyn, from a calibrated stroke-volume signal, or from arterial morphology measured with a higher-resolution channel. Thus, the conclusion is specific to waveform-derived surrogates of the kind that can be extracted from archived registries. Modal class assignment does not propagate classification uncertainty; the three bias-adjusted three-step estimators presented the same answer, yet all three depend on the adequacy of the latent-class model, whose information criteria decreased monotonically so that the class number was set by the prespecified range of one to five classes and the minimum-class constraint rather than by fit. The selected solution lies at the upper limit of that range, and we did not examine solutions with more classes. The class structure was also sensitive to cohort composition: restricting the cohort to adults changed the selected solution from four to five classes and redistributed the smaller classes, which illustrates that latent-class phenotypes of this kind are descriptive summaries rather than stable biological entities. Because hypotension burden may itself mediate any effect of waveform morphology, the absence of incremental value beyond that term does not exclude an association between morphology and AKI in models that omit it. The expanded clinical model had 5.5 events per parameter and is reported as a sensitivity reference model, not as a prediction tool; its incremental comparisons were bootstrap-corrected. Finally, the analysis is retrospective and observational.

### Future Research Directions

Three potential directions for future research may resolve what this study cannot. First, the same questions explored in this study should be asked with device-derived dP/dtmax and Eadyn and a calibrated stroke-volume signal, prospectively recorded at full amplitude resolution, so that measurement error no longer limits the inference; thus a modification could be avoided altogether. Second, higher-risk populations, such as cardiac surgery patients and those with chronic kidney disease, in which renal reserve is low and hemodynamic perturbation larger, are those in which a morphological signal, if it exists, is most likely to be detectable and useful. A separate study focusing on these populations might obtain different results. Third, because waveform morphology predicts impending hypotension, its clinical value may lie in prevention rather than prognosis: trials in which waveform-guided hemodynamic management is the intervention and AKI the outcome, with intraoperative time-updated models and a mediation framework that treats hypotension as the intermediate, would test the mechanism directly.

## 5. Conclusions

In a large registry cohort of adult surgical patients, intraoperative arterial waveform morphology did not improve prediction of postoperative AKI beyond a simple model based on intraoperative hypotension burden and baseline characteristics, and it did not improve prediction beyond a richer clinical model either. The prognostic signal for AKI resides in the achieved pressure and its cumulative burden, together with the patient’s baseline and procedural risk, not in the shape of the waveform that generates the pressure. Trajectory-class membership was weakly associated with AKI, but the association was carried by the most unstable operations and conferred no gain in discrimination or net benefit. With an adequately powered primary test, bias-adjusted class linkage, optimism correction, and alternative time axes, these data exclude an incremental gain in discrimination greater than approximately 0.02–0.03 AUC units for the waveform-derived surrogates studied, but cannot exclude a smaller one or a signal from device-derived indices. Prospective evaluation in higher-risk populations, using absolute rather than estimator-dependent measures of contractility, would be needed before waveform morphology could be excluded as a risk marker across the broader surgical spectrum.

## Figures and Tables

**Table 1:** Cohort characteristics, waveform features, and intraoperative hypotension burden.

Parameters (Median, IQR)	Value (n = 2587)
***Patient characteristics***	
Age (years)	62 (53–70)
Sex (%)	
Female	40.9
Male	59.1
BMI (kg/m^2^)	23.0 (20.9–25.1)
ASA Class	2 (2–2)
ASA class ≥ 3 (%)	14.8
Hypertension (%)	33.6
Diabetes mellitus (%)	13.0
Emergency surgery (%)	11.2
Baseline eGFR (mL/min/1.73 m^2^)	95.2 (83.6–104.0)
Baseline creatinine (mg/dL)	0.80 (0.68–0.96)
Postoperative AKI (any stage), n (%)	143 (5.5)
KDIGO stage 1/2/3, n	93/25/25
Intensive-care admission (%)	33.6
In-hospital death (%)	0.7
***Surgical characteristics and waveform features***	
Operative duration (min)	160 (105–240)
Crystalloid (mL)	1150 (650–1900)
Any vasopressor bolus (%)	62.8
Red-cell transfusion (%)	9.1
Usable signal fraction	0.98 (0.96–0.99)
Heart rate (bpm)	72 (63–81)
Mean arterial pressure (mmHg)	83 (76–90)
Pulse pressure (mmHg)	54 (48–61)
dP/dtmax (mmHg/s)	737 (621–884)
τ (s)	0.24 (0.21–0.28)
Dynamic arterial elastance	0.28 (0.21–0.40)
***Intraoperative hypotension burden***	
TWA < 65 (mmHg·min)	10.6 (0.4–67.7)
Minimum MAP (mmHg)	52 (45–60)

**IQR:** interquartile range, **BMI:** body mass index, **ASA:** American Society of Anesthesiologists, **eGFR:** estimated glomerular filtration rate, **KDIGO:** Kidney Disease: Improving Global Outcomes, **MAP:** mean arterial pressure, **dP/dtmax:** maximum rate of pressure change over time, **τ:** late-diastolic pressure-decay descriptor (tau), **TWA < 65:** time-weighted area under a MAP of 65 mmHg, **AKI:** acute kidney injury.

**Table 2:** Latent-class model selection for trajectories.

Classes	BIC	aBIC	ICL-BIC	Entropy	Smallest Class (%)
***dP/dtmax***					
1	664,289.97	664,264.56	664,289.97	—	100
2	661,275.37	661,234.07	662,457.51	0.670	48.0
3	659,674.54	659,617.35	660,816.34	0.799	15.4
4	658,614.26	658,541.18	659,781.08	0.837	9.3
5	657,912.88	657,823.91	659,199.04	0.846	6.5
***τ (log scale)***					
1	−13,599.75	−13,625.17	−13,599.75	—	100
2	−16,444.99	−16,486.29	−16,413.49	0.991	3.1
3	−18,313.38	−18,370.57	−18,241.07	0.987	2.0
4	−19,425.10	−19,498.17	−18,746.26	0.905	1.9
5	−20,302.38	−20,391.35	−19,600.35	0.916	1.6

**BIC:** Bayesian information criterion, **dP/dtmax:** maximum rate of pressure change over time, **τ:** late-diastolic pressure-decay descriptor (tau), **aBIC:** sample-size-adjusted BIC, **ICL-BIC:** integrated-completed-likelihood BIC. Entropy is the relative entropy (0–1). The selected solution (five classes for dP/dtmax, the upper limit of the prespecified range) is the lowest-BIC solution in which every class contains at least 5% of cases; for τ, no multi-class solution satisfied this constraint.

**Table 3:** Trajectory phenotypes for the five-class solution.

Class	n	AKI, n (%)	TWA < 65 (Median, mmHg·min)	dP/dtmax Start (mmHg/s)	dP/dtmax End (mmHg/s)
1	247	18 (7.3)	10.2	786	914
2	167	11 (6.6)	11.4	805	836
3	1683	77 (4.6)	9.2	728	763
4	275	28 (10.2)	21.8	1034	974
5	215	9 (4.2)	10.8	929	1170

**AKI:** acute kidney injury, **TWA < 65:** time-weighted area under MAP 65 mmHg, **dP/dtmax:** maximum rate of pressure change over time. dP/dtmax start and end are defined by rescaling each case’s operative window to a common axis divided into 20 equal bins; the two values are the class-mean dP/dtmax in the first and last bin. Classes are numbered as in the latent-class output and are listed in the same order throughout the text.

**Table 4:** Base logistic model for postoperative acute kidney injury.

Predictors	Odds Ratio (95% CI)	*p*
Hypotension burden, log(1 + TWA < 65)	1.439 (1.324–1.563)	<0.001
Age, per year	0.979 (0.966–0.993)	0.003
Male sex	2.022 (1.363–3.001)	<0.001
Baseline eGFR, per mL/min/1.73 m^2^	0.991 (0.983–1.000)	0.061

**CI:** confidence interval, **eGFR:** estimated glomerular filtration rate, **TWA < 65:** time-weighted area under a MAP of 65 mmHg. Hypotension burden is modeled as log(1 + TWA < 65). The model has an AUC of 0.725 (95% CI 0.680–0.770) for discrimination; optimism-corrected AUC 0.718, calibration slope 0.97, Brier score 0.049 (n = 2587, 143 events).

**Table 5:** Incremental value of waveform morphology added to the base model.

Predictor Added to Base Model	Odds Ratio	ΔAUC (95% CI)	Optimism Corrected ΔAUC	IDI (95% CI)	LR *p*	q (BH)
***Latent-class trajectory models***						
dP/dtmax trajectory class ^a^	—	0.008 (−0.005–0.021)	0.002	0.0091 (0.0033–0.0151)	0.02	0.051
τ trajectory class (5 classes, unselected) ^a^	—	−0.002 (−0.008–0.003)	−0.010	0.0023 (0.0006–0.0039)	0.83	0.83
***Case-level summaries***						
Dynamic arterial elastance ^b^	2.51	0.005 (−0.004–0.013)	0.003	0.0015 (−0.0015–0.0051)	0.07	0.088
Pulse-pressure variation ^b^	1.019	0.008 (−0.001–0.017)	0.007	0.0006 (−0.0020–0.0035)	0.05	0.08
Stroke-volume variation ^b^	1.009	0.004 (−0.001–0.009)	0.002	−0.0004 (−0.0016–0.0008)	0.21	0.23
dP/dtmax (median)	1.001	0.004 (−0.008–0.016)	0.002	0.0061 (−0.0004–0.0137)	0.01	0.041
τ (median)	0.059	0.005 (−0.002–0.012)	0.004	0.0016 (−0.0020–0.0051)	0.06	0.085
MAP, coefficient of variation	165.4	0.003 (−0.005–0.011)	0.001	0.0041 (0.0005–0.0079)	0.04	0.08
Pulse pressure, coefficient of variation	49.4	0.007 (−0.004–0.018)	0.006	0.0072 (0.0020–0.0134)	0	0.032
dP/dtmax, coefficient of variation	9.904	0.003 (−0.006–0.011)	0.002	0.0063 (0.0018–0.0118)	0.01	0.041
***Absolute-time representations (sensitivity analyses)***						
DTW k-medoids cluster (k = 3) ^a^	—	0.006 (−0.004–0.017)	0.003	0.0045 (0.0006–0.0083)	0.04	—
Group-based trajectory class (K = 5) ^a^	—	0.012 (−0.003–0.028)	0.006	0.0098 (0.0032–0.0172)	0.01	—

**AUC:** area under the curve, **IDI:** integrated discrimination improvement, **LR:** likelihood ratio, **BH:** Benjamini–Hochberg false-discovery-rate-adjusted *p*, **DTW:** dynamic time warping, **dP/dtmax:** maximum rate of pressure change over time, **τ:** late-diastolic pressure-decay descriptor (tau), **MAP:** mean arterial pressure. Base model consisted of log(1 + TWA < 65), age, sex, and baseline estimated glomerular filtration rate. Each row adds the listed predictor to the base model; odds ratios are per unit of the predictor. Confidence intervals for ΔAUC are DeLong intervals, and those for the IDI are bootstrap percentile intervals (1000 replications); optimism-corrected ΔAUC is from Harrell’s bootstrap (1000 resamples). The likelihood-ratio test is the primary test of incremental value. ^a^ Class or cluster membership is a multi-level factor, for which a single odds ratio is not defined. ^b^ Waveform-derived surrogate, not a device-derived measurement.

**Table 6:** Incremental value of waveform morphology across alternative specifications of the reference model.

Reference Model	Model Parameters	Base AUC (95% CI)	Largest ΔAUC (Feature, PPV)	Largest Upper 95% Limit of ΔAUC	Smallest LR *p* (Feature)
Log(1 + TWA < 65) + age + sex + eGFR (base)	4	0.725 (0.680–0.770)	0.008	0.021 (trajectory class)	0.003 (PP CV)
Restricted cubic spline (4 knots) for log-burden	6	0.730 (0.685–0.774)	0.011	0.024 (trajectory class)	0.008 (median dP/dtmax)
Splines (3 knots) for log-burden and age	6	0.727 (0.682–0.773)	0.013	0.028 (trajectory class)	0.005 (median dP/dtmax)
Base + log(1 + TWA < 55)	5	0.727 (0.683–0.772)	0.010	0.023 (trajectory class)	0.011 (median dP/dtmax)
Log minutes MAP < 60 in place of TWA < 65	4	0.716 (0.670–0.762)	0.013	0.027 (PPV)	0.004 (PP CV)
Untransformed TWA < 65 (poorly fitting) ^a^	4	0.699 (0.653–0.746)	0.022	0.044 (trajectory class)	<0.001 (MAP CV)
Base + crystalloid rate, colloid, vasopressor	7	0.752 (0.709–0.794)	0.009	0.020 (median dP/dtmax)	0.004 (median dP/dtmax)
Expanded clinical model (22 covariates)	26	0.837 (0.804–0.870)	0.005	0.012 (PPV)	0.023 (PPV)

Each row refits the reference model with the listed specification, then adds each of the nine prespecified and exploratory morphology representations (dP/dtmax trajectory class, Eadyn, PPV, SVV, medians of dP/dtmax and τ, coefficients of variation of MAP, pulse pressure, and dP/dtmax). ^a^ Shown as an example of a deliberately mis-specified reference model: the poorer the reference model, the larger the apparent contribution of morphology. **AUC:** area under the curve, **CV:** coefficient of variation, **eGFR:** estimated glomerular filtration rate, **LR:** likelihood ratio, **PP:** pulse pressure, **PPV:** pulse-pressure variation, **TWA:** time-weighted area.

## Data Availability

The VitalDB registry is publicly available at https://vitaldb.net. The complete analysis code (cohort derivation, waveform feature extraction and quality control, latent-class trajectory modeling, outcome models, and all sensitivity analyses), together with the derived case-level and per-minute feature tables, the class assignments and a specification of the software environment, is openly available in the Harvard Dataverse at https://doi.org/10.7910/DVN/WW7T2W.

## Supplementary Materials

The following supporting information can be downloaded at: https://www.mdpi.com/article/10.3390/jcm15176871/s1, Table S1: Comparison of the analysis cohort with adult waveform cases excluded at each step; Table S2: Expanded clinical reference model; Table S3: Bias-adjusted three-step estimates of the class–outcome association; Table S4: Characteristics of absolute-time clusters and classes and their incremental value on the base and expanded reference models; Table S5: Incremental statistics for every morphology representation; Table S6: Simulation-based power and minimum detectable ΔAUC and IDI; Table S7: Incremental value for KDIGO stage ≥2; Table S8: Incremental value across all alternative reference models; Figure S1: Calibration and decision curves; Figure S2: Absolute-time trajectory classes and clusters; Figure S3: Dose–response of hypotension burden.

## Abbreviations

The following abbreviations are used in this manuscript:

aBIC	Sample-size-adjusted Bayesian information criterion
AKI	Acute kidney injury
APPA	Average posterior probability of assignment
ASA	American Society of Anesthesiologists
AUC	Area under the receiver operating characteristic curve
BCH	Bolck–Croon–Hagenaars correction
BH	Benjamini–Hochberg
BIC	Bayesian information criterion
BMI	Body mass index
CI	Confidence interval
CKD-EPI	Chronic Kidney Disease Epidemiology Collaboration
CV	Coefficient of variation
dP/dtmax	Maximum rate of arterial pressure rise
DTW	Dynamic time warping
Eadyn	Dynamic arterial elastance
eGFR	Estimated glomerular filtration rate
ESRD	End-stage renal disease
FDR	False discovery rate
GBTM	Group-based trajectory model
ICL-BIC	Integrated completed likelihood Bayesian information criterion
ICU	Intensive care unit
IDI	Integrated discrimination improvement
IQR	Interquartile range
KDIGO	Kidney Disease: Improving Global Outcomes
LR	Likelihood ratio
MAP	Mean arterial pressure
OCC	Odds of correct classification
OR	Odds ratio
PPV	Pulse-pressure variation
QC	Quality control
RCS	Restricted cubic spline
ROC	Receiver operating characteristic
SNUADC/ART	Seoul National University Anesthesia Data Collector, invasive arterial pressure channel
STROBE	Strengthening the Reporting of Observational Studies in Epidemiology
SVV	Stroke-volume variation
TWA < 65	Time-weighted area under a mean arterial pressure of 65 mmHg
VitalDB	Vital Signs Database
τ	Diastolic pressure-decay time constant (empirical late-diastolic decay descriptor)
