# Supplementary material for: Intraoperative Arterial Waveform Morphology Trajectories and Prediction of Postoperative Acute Kidney Injury: A Latent-Class Analysis of the VitalDB Registry
Source: J Clin Med. 2026 Sep 4;15(17):6871. doi: 10.3390/jcm15176871 (PMC13566423; doi:10.3390/jcm15176871)
Supplement: Supplementary file 1 [file jcm-15-06871-s001.zip › jcm-4514602-supplementary.pdf]

Supplementary Materials

Table S1. Comparison of the analysis cohort with adult waveform cases excluded at each step of the derivation

Characteristic	Analysed (n = 2587)	Excluded, any reason (n = 989)	No baseline Cr (n = 264)	No postoperative Cr ≤7 d (n = 523)	ESRD (n = 115)	Extraction / QC (n = 87)	p (analyzed vs excluded)
Age, years	62 (53–70)	57 (45–65)	59 (48–66)	56 (44–65)	52 (42–60)	62 (56–73)	<0.001
Male sex, %	59.1	47.4	56.8	39.0	59.1	54.0	<0.001
BMI, kg/m²	23.0 (20.9–25.1)	22.9 (20.5–25.3)	22.8 (20.6–25.0)	23.2 (20.9–25.7)	21.1 (19.1–23.6)	22.6 (20.6–24.8)	0.41
ASA class ≥3, %	14.8	20.2	13.5	14.3	62.6	12.6	0.011
Emergency surgery, %	11.2	18.8	26.9	10.7	39.1	16.1	<0.001
Hypertension, %	33.6	33.2	29.9	26.0	70.4	36.8	
Diabetes mellitus, %	13.0	10.1	10.2	7.8	14.8	17.2	
Baseline creatinine, mg/dL	0.8 (0.7–1.0)	0.8 (0.7–1.1)	—	0.7 (0.6–0.9)	7.9 (6.3–10.1)	0.8 (0.6–0.9)	
Baseline eGFR, mL/min/1.73 m²	95 (84–104)	97 (70–107)	—	101 (89–110)	7 (5–9)	97 (87–105)	0.42
Operative duration, min	160 (105–240)	113 (70–190)	120 (80–201)	90 (57–155)	230 (172–265)	103 (61–196)	<0.001
Crystalloid, mL	1150 (650–1900)	800 (400–1551)	800 (450–1500)	650 (400–1188)	2000 (1500–2500)	800 (350–1488)	<0.001
Any RBC transfusion, %	9.1	8.7	10.2	6.1	17.4	8.0	0.74
ICU admission, %	33.6	23.5	37.5	12.4	45.2	18.4	<0.001
In-hospital death, %	0.7	1.8	1.5	2.1	0.0	3.4	
Transplantation, %	5.8	14.9	11.0	2.9	80.0	12.6	
Hepatic, %	8.7	2.7	4.5	2.3	0.9	2.3	
Biliary/Pancreas, %	10.5	9.8	6.4	14.0	2.6	4.6	
Colorectal, %	10.7	12.9	7.2	10.9	5.2	52.9	
Stomach, %	15.3	4.8	3.0	5.0	1.7	12.6	
Major resection, %	15.7	14.6	15.9	18.9	0.0	3.4	
Minor resection, %	15.1	11.5	24.2	9.4	0.0	1.1	
Vascular, %	2.6	3.3	2.7	4.2	1.7	2.3	
Others, %	15.7	25.5	25.0	32.5	7.8	8.0	

Values are median (interquartile range) or %. 'Excluded, any reason' pools the four exclusion strata. p-values: Mann–Whitney U test for continuous variables, χ^2 test for proportions (analyzed vs excluded, any reason); procedure-type distribution differed overall (χ^2 $p < 0.001$). Among adult, non-ESRD waveform cases with a baseline creatinine (n = 3185), a postoperative creatinine was measured within 7 days in 84.0%.

Table S2. Expanded clinical reference model for postoperative AKI

Predictor	Odds ratio (95% CI)	p
Hypotension burden, log(1 + TWA<65)	1.18 (1.05–1.32)	0.004
Age, per year	0.99 (0.98–1.01)	0.42
Male sex	1.84 (1.20–2.81)	0.005
Baseline eGFR, per mL/min/1.73 m²	1.00 (0.99–1.01)	0.92
Emergency surgery	1.57 (0.93–2.64)	0.089
ASA class ≥3	2.92 (1.90–4.49)	<0.001
Body-mass index, per kg/m²	1.01 (0.95–1.07)	0.77
Hypertension	1.68 (1.08–2.60)	0.020
Diabetes mellitus	1.07 (0.64–1.79)	0.80
Operative duration, per log-minute	1.33 (0.84–2.09)	0.22
Crystalloid, per L/h	0.85 (0.48–1.50)	0.58
Any colloid	1.25 (0.71–2.20)	0.43
Blood loss, per log-mL	1.12 (0.86–1.47)	0.39
Blood loss not recorded	0.98 (0.51–1.86)	0.94
Any red-cell transfusion	1.92 (1.06–3.44)	0.030
Any phenylephrine bolus	1.02 (0.65–1.59)	0.93
Any ephedrine bolus	0.80 (0.51–1.24)	0.31
Any epinephrine bolus	0.73 (0.31–1.72)	0.48
Procedure: biliary/pancreatic (vs others)	0.69 (0.35–1.35)	0.28
Procedure: colorectal	0.49 (0.23–1.02)	0.055
Procedure: hepatic	0.52 (0.23–1.13)	0.099
Procedure: major resection	0.42 (0.20–0.88)	0.021
Procedure: minor resection	0.32 (0.12–0.80)	0.015
Procedure: gastric	0.12 (0.03–0.40)	<0.001
Procedure: transplantation	1.46 (0.67–3.17)	0.35
Procedure: vascular	0.27 (0.10–0.75)	0.012

Logistic regression in the analysis cohort (n = 2587, 143 events; 5.5 events per parameter). AUC 0.837 (95% CI 0.804–0.870); likelihood-ratio test versus the base model χ^2 = 140.3, 22 df, p < 0.001. This model serves as the sensitivity reference model for the incremental comparisons (Table 6 of the main text).

Table S3. Bias-adjusted three-step estimates of the association between dP/dtmax latent class and AKI

Estimator	Class 2 vs 1, OR (95% CI)	Class 3 vs 1	Class 4 vs 1	Class 5 vs 1	χ^2 (4 df)	p	ΔAUC
Modal assignment	0.92 (0.41–2.05)	0.59 (0.34–1.02)	1.24 (0.65–2.36)	0.56 (0.24–1.31)	11.64	0.020	+0.0079
Posterior probabilities as covariates	0.98 (0.40–2.41)	0.57 (0.31–1.06)	1.29 (0.63–2.63)	0.44 (0.16–1.18)	11.06	0.026	+0.0077
Pseudo-class draws (M = 200)	0.99 (0.42–2.31)	0.66 (0.37–1.19)	1.18 (0.59–2.35)	0.55 (0.22–1.41)	8.61	0.071	+0.0065 (range +0.0003 to +0.0158)
BCH-weighted (cluster-robust)	0.92 (0.38–2.25)	0.52 (0.27–0.98)	1.30 (0.65–2.59)	0.49 (0.17–1.38)	12.53	0.014	—

Five-class dP/dtmax solution refitted in the adult cohort. All models adjust for log(1 + TWA<65), age, sex and baseline eGFR. Modal assignment: each case assigned to its most probable class (likelihood-ratio test). Posterior probabilities: the probabilities of classes 2–5 entered as continuous covariates (likelihood-ratio test). Pseudo-class draws: class membership drawn 200 times from each case's posterior distribution, estimates combined by Rubin's rules; the χ^2 is the mean likelihood-ratio statistic across draws and the p-value refers to it. BCH: Bolck–Croon–Hagenaars weighting with the inverse of the estimated classification-error matrix (diagonal 0.85, 0.82, 0.95, 0.78, 0.84) and cluster-robust standard errors; Wald test. Average posterior probability of assignment by class 0.89, 0.87, 0.92, 0.85, 0.86; relative entropy 0.846; 12.5% of cases had a maximum posterior probability below 0.70. Class 3 is the dominant flat class (n = 1683); the association is carried by its lower adjusted risk relative to the non-flat classes.

Table S4. Absolute-time clusters and classes of the dP/dtmax series

Group	n	AKI (%)	Median dP/dtmax (mmHg/s)	Pulse pressure (mmHg)	Age (y)	ASA ≥3 (%)	Emergency (%)	Transfusion (%)	TWA<65 (mmHg·min)	Duration (min)
DTW cluster 1	1339	4.3	740	53	61	14	11	8	9.4	160
DTW cluster 2	609	7.4	1014	65	66	18	15	11	12.2	165
DTW cluster 3	639	6.4	539	45	60	14	8	9	11.7	160
GBTM class 1	470	6.6	516	44	60	15	9	10	11.5	160
GBTM class 2	626	3.8	655	50	59	13	10	7	7.6	160
GBTM class 3	552	6.5	966	63	65	15	12	10	12.9	170
GBTM class 4	801	4.5	776	55	62	14	11	9	10.1	161
GBTM class 5	138	11.6	1264	75	71	28	23	15	14.0	150
Normalized-time class 1	247	7.3	919	60	64	15	13	11	10.2	145
Normalized-time class 2	167	6.6	887	60	66	14	13	8	11.4	124
Normalized-time class 3	1683	4.6	688	52	60	14	10	8	9.2	165
Normalized-time class 4	275	10.2	778	55	63	20	14	16	21.8	185
Normalized-time class 5	215	4.2	855	59	65	15	17	7	10.8	160

DTW: dynamic-time-warping k-medoids on 5-min medians of dP/dtmax in absolute time (Sakoe–Chiba band 60 min; silhouette width 0.36 for k = 2, 0.26 for k = 3, 0.14 for k = 4). GBTM: group-based trajectory model on absolute minutes (natural cubic spline, 3 df; EM algorithm; BIC decreased monotonically from K = 1 to 5, smallest class 5.3% at K = 5). Incremental value on the base model: DTW k = 3, ΔAUC 0.006 (95% CI −0.004 to 0.017), LR χ^2 6.62 (2 df), p = 0.037, IDI 0.0045, optimism-corrected ΔAUC 0.003; DTW k = 4, ΔAUC 0.007 (−0.005 to 0.018), LR p = 0.053; GBTM K = 5, ΔAUC 0.012 (−0.003 to 0.028), LR χ^2 13.41 (4 df), p = 0.009, IDI 0.0098 (0.0032–0.0172), optimism-corrected ΔAUC 0.006.

Table S5. Incremental statistics for every morphology representation added to the base model

Representation	AUC	ΔAUC (DeLong 95% CI)	DeLong p	IDI (bootstrap 95% CI)	LR χ^2 (df)	LR p	q (BH)	Corrected ΔAUC	Corrected IDI	Corrected slope	Brier	Max decile dev.
dP/dtmax trajectory class (lcmm, normalised time)	0.733	+0.0079 (-0.0052, +0.0210)	0.24	+0.0091 (+0.0033, +0.0151)	11.64 (4)	0.020	0.051	+0.0015	+0.0064	0.939	0.0485	0.053
tau trajectory class (lcmm, 5-class, not selected)	0.723	-0.0022 (-0.0076, +0.0032)	0.43	+0.0023 (+0.0006, +0.0039)	1.47 (4)	0.83	0.833	-0.0103	-0.0004	0.874	0.0488	0.025
Dynamic arterial elastance	0.730	+0.0048 (-0.0036, +0.0132)	0.27	+0.0015 (-0.0015, +0.0051)	3.27 (1)	0.070	0.088	+0.0030	+0.0006	0.960	0.0489	0.046
Pulse-pressure variation	0.733	+0.0083 (-0.0008, +0.0174)	0.074	+0.0006 (-0.0020, +0.0035)	3.91 (1)	0.048	0.080	+0.0067	-0.0002	0.958	0.0491	0.026
Stroke-volume variation	0.729	+0.0040 (-0.0014, +0.0094)	0.14	-0.0004 (-0.0016, +0.0008)	1.56 (1)	0.21	0.235	+0.0022	-0.0008	0.960	0.0491	0.015
dP/dtmax (median)	0.729	+0.0041 (-0.0081, +0.0164)	0.51	+0.0061 (-0.0004, +0.0137)	6.80 (1)	0.009	0.041	+0.0021	+0.0046	0.961	0.0487	0.041
tau (median)	0.730	+0.0054 (-0.0016, +0.0123)	0.13	+0.0016 (-0.0020, +0.0051)	3.55 (1)	0.060	0.085	+0.0037	+0.0007	0.962	0.0490	0.023
MAP, coefficient of variation	0.728	+0.0029 (-0.0051, +0.0109)	0.48	+0.0041 (+0.0005, +0.0079)	4.14 (1)	0.042	0.080	+0.0012	+0.0032	0.963	0.0488	0.018
Pulse pressure, coefficient of variation	0.733	+0.0074 (-0.0035, +0.0184)	0.18	+0.0072 (+0.0020, +0.0134)	8.71 (1)	0.003	0.032	+0.0061	+0.0065	0.965	0.0487	0.025
dP/dtmax, coefficient of variation	0.728	+0.0026 (-0.0061, +0.0113)	0.55	+0.0063 (+0.0018, +0.0118)	6.28 (1)	0.012	0.041	+0.0015	+0.0055	0.965	0.0487	0.022
DTW cluster, absolute time (k=3)	0.731	+0.0063 (-0.0044, +0.0170)	0.25	+0.0045 (+0.0006, +0.0083)	6.62 (2)	0.037	—	+0.0033	+0.0031	0.956	0.0488	0.032
GBTM class, absolute time (K=5)	0.737	+0.0123 (-0.0029, +0.0275)	0.11	+0.0098 (+0.0032, +0.0172)	13.41 (4)	0.009	—	+0.0061	+0.0070	0.941	0.0485	0.024

Base model: AUC 0.725 (0.680–0.770), optimism-corrected 0.718, calibration slope 0.972, calibration-in-the-large 0.000, Brier 0.0490, maximum decile deviation 0.021. Optimism corrections by Harrell's bootstrap (1000 resamples); IDI confidence intervals by bootstrap percentiles (1000 replications); q: Benjamini–Hochberg adjusted p over the ten prespecified and exploratory tests. Decision-curve analysis (thresholds 2–30%): net benefit of the base model 0.0197 at a threshold of 5%, 0.0096 at 10%, 0.0071 at 15% and 0.0042 at 20%; the largest absolute difference between any extended model and the base model was 0.0045 (GBTM class at 10%) and 0.0042 for the dP/dtmax trajectory class.

Table S6. Simulation-based power and minimum detectable increment at the actual sample size

Marker log-odds ratio per SD (γ)	Expected ΔAUC	Expected IDI	Power, LR test (%)	Power, DeLong test (%)
0.00	0.0010	0.0006	5	0
0.10	0.0021	0.0013	20	1
0.15	0.0037	0.0024	41	3
0.20	0.0056	0.0037	61	7
0.25	0.0083	0.0056	79	17
0.30	0.0116	0.0077	94	24
0.35	0.0145	0.0101	97	35
0.40	0.0210	0.0141	99	62
0.50	0.0293	0.0203	100	83

Outcomes regenerated 300 times per γ from the fitted base linear predictor plus γ × a standard-normal marker independent of the base covariates, with the intercept recentered to the observed event rate (5.5%); n = 2587. By linear interpolation, the likelihood-ratio test had 80% power at ΔAUC = 0.0086 (IDI 0.0058) and 90% power at ΔAUC = 0.0107 (IDI 0.0071). The DeLong test reached 80% power at ΔAUC = 0.028.

Table S7. Incremental value of waveform morphology for KDIGO stage ≥2 AKI

Predictor added to base model	Odds ratio	Wald p	ΔAUC (95% CI)	DeLong p	IDI	LR p
dP/dtmax trajectory class	—	—	+0.0116 (-0.0127, +0.0358)	0.35	+0.0109	0.064
Dynamic arterial elastance	4.167	0.061	+0.0084 (-0.0111, +0.0278)	0.40	+0.0031	0.073
Pulse-pressure variation	1.020	0.15	+0.0071 (-0.0060, +0.0202)	0.29	+0.0001	0.20
Stroke-volume variation	0.995	0.76	-0.0008 (-0.0043, +0.0028)	0.68	+0.0006	0.75
dP/dtmax (median)	1.002	<0.001	+0.0115 (-0.0174, +0.0404)	0.43	+0.0199	<0.001
τ (median)	0.046	0.23	+0.0093 (-0.0006, +0.0192)	0.067	-0.0003	0.20
MAP, coefficient of variation	1079.265	0.073	+0.0029 (-0.0147, +0.0204)	0.75	+0.0060	0.080
Pulse pressure, coefficient of variation	58.251	0.042	+0.0156 (-0.0009, +0.0321)	0.064	+0.0018	0.051
dP/dtmax, coefficient of variation	4.891	0.27	+0.0057 (-0.0020, +0.0134)	0.15	+0.0004	0.29

Base model for stage ≥2 AKI: log(1 + TWA<65) OR 1.545 (1.341–1.778), age 0.967 per year (0.947–0.988), male sex 1.21 (0.66–2.21), eGFR 0.981 per mL/min/1.73 m² (0.969–0.993); AUC 0.755 (0.680–0.830).

Table S8. Incremental value of waveform morphology across all alternative specifications of the reference model

Reference model (all include age, sex, eGFR unless stated)	Parameters	Base AUC (95% CI)	AIC	Largest ΔAUC (feature)	Largest upper 95% limit	Smallest LR p (feature)
RCS3 burden + RCS3 age	6	0.727 (0.682–0.773)	1004.4	0.0125 (Pulse-pressure variation)	0.0278	0.005 (dP/dtmax (median))
RCS4 burden + RCS4 age	8	0.736 (0.691–0.781)	1001.6	0.0103 (Pulse-pressure variation)	0.0249	0.004 (dP/dtmax (median))
RCS4 burden, linear age	6	0.730 (0.685–0.774)	1003.7	0.0107 (Pulse-pressure variation)	0.0244	0.008 (dP/dtmax (median))
expanded clinical base	26	0.837 (0.804–0.870)	915.2	0.0050 (Pulse-pressure variation)	0.0117	0.023 (Pulse-pressure variation)
linear burden, RCS4 age	6	0.731 (0.687–0.776)	1008.8	0.0091 (dP/dtmax trajectory class)	0.0226	0.002 (Pulse pressure, coefficient of variation)
log TWA<55 (per-minute)	4	0.704 (0.658–0.750)	1018.4	0.0167 (Pulse-pressure variation)	0.0373	0.006 (Pulse pressure, coefficient of variation)
log TWA<60 (per-minute)	4	0.712 (0.666–0.758)	1014.4	0.0127 (Pulse-pressure variation)	0.0267	0.005 (Pulse pressure, coefficient of variation)
log TWA<65 (per-minute)	4	0.719 (0.673–0.764)	1015.4	0.0116 (Pulse-pressure variation)	0.0243	0.002 (Pulse pressure, coefficient of variation)
log TWA<65 + log TWA<55	5	0.727 (0.683–0.772)	1006.4	0.0104 (Pulse-pressure variation)	0.0225	0.011 (dP/dtmax (median))
log TWA<65 + log TWA<55 + min MAP	6	0.728 (0.683–0.772)	1008.4	0.0104 (Pulse-pressure variation)	0.0223	0.011 (dP/dtmax (median))
log TWA<70 (per-minute)	4	0.715 (0.669–0.761)	1024.5	0.0111 (Pulse pressure, coefficient of variation)	0.0252	<0.001 (Pulse pressure, coefficient of variation)
log minutes MAP<55	4	0.706 (0.660–0.752)	1013.6	0.0172 (Pulse-pressure variation)	0.0367	0.007 (Dynamic arterial elastance)
log minutes MAP<60	4	0.716 (0.670–0.762)	1010.0	0.0127 (Pulse-pressure variation)	0.0270	0.004 (Pulse pressure, coefficient of variation)
log minutes MAP<65	4	0.723 (0.678–0.768)	1013.1	0.0115 (Pulse-pressure variation)	0.0236	<0.001 (Pulse pressure, coefficient of variation)
log minutes MAP<70	4	0.713 (0.668–0.759)	1027.2	0.0136 (Pulse pressure, coefficient of variation)	0.0279	<0.001 (Pulse pressure, coefficient of variation)
minimum MAP	4	0.704 (0.660–0.749)	1034.5	0.0167 (Pulse pressure, coefficient of variation)	0.0324	<0.001 (Pulse pressure, coefficient of variation)
original (log TWA<65)	4	0.725 (0.680–0.770)	1011.4	0.0083 (Pulse-pressure variation)	0.0210	0.003 (Pulse pressure, coefficient of variation)
original + fluids + vasopressors	7	0.752 (0.709–0.794)	991.5	0.0088 (Pulse-pressure variation)	0.0195	0.004 (dP/dtmax (median))
raw TWA<65 (untransformed)	4	0.699 (0.653–0.746)	1042.1	0.0224 (Pulse-pressure variation)	0.0441	<0.001 (MAP, coefficient of variation)
sqrt TWA<65	4	0.732 (0.688–0.777)	1006.6	0.0115 (Pulse-pressure variation)	0.0257	0.007 (dP/dtmax (median))

For each reference model the nine prespecified and exploratory morphology representations were added in turn. The expanded clinical base is the model of Table S2; 'original + fluids + vasopressors' adds crystalloid rate, any colloid, and any vasopressor bolus to the base model.

Figure S1. Calibration and decision curves of the base model and of extended models

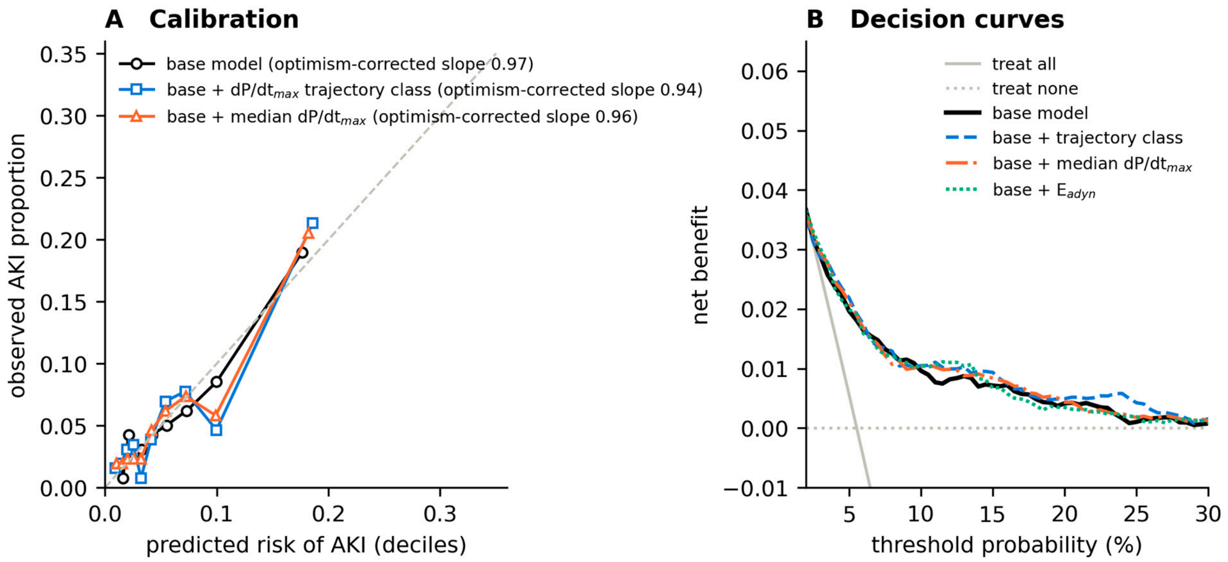

(A) Observed AKI proportion against mean predicted risk in deciles of predicted risk for the base model and for the base model plus the dP/dtmax trajectory class or the median dP/dtmax; dashed line, perfect calibration. (B) Decision curves (net benefit against threshold probability) for treat-all, treat-none, the base model and three extended models.

Figure S2. Absolute-time representations of the intraoperative dP/dtmax series

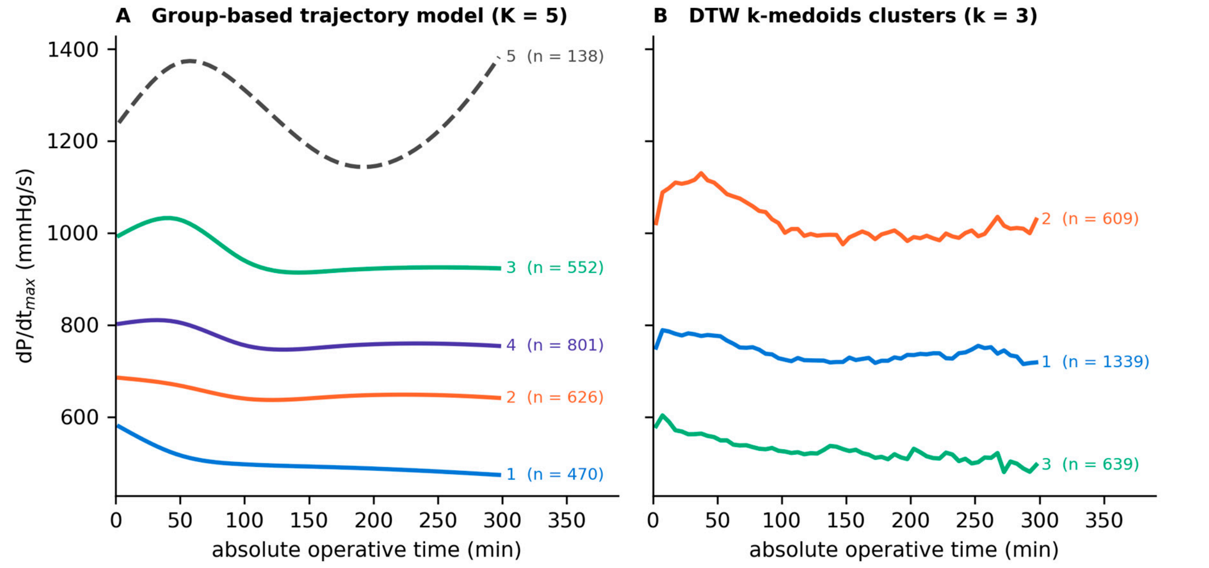

Class/cluster means of per-case dP/dt$_{max}$ (5-min medians) plotted against absolute operative time; numbers at the right identify the class and its size.

(A) Class-mean trajectories of the five-class group-based trajectory model fitted on absolute operative minutes (natural cubic spline, 3 df; EM algorithm); (B) cluster means of the three-cluster dynamic-time-warping k-medoids solution. Both separate cases by the level rather than the shape of dP/dtmax.

Figure S3. Dose–response of intraoperative hypotension burden

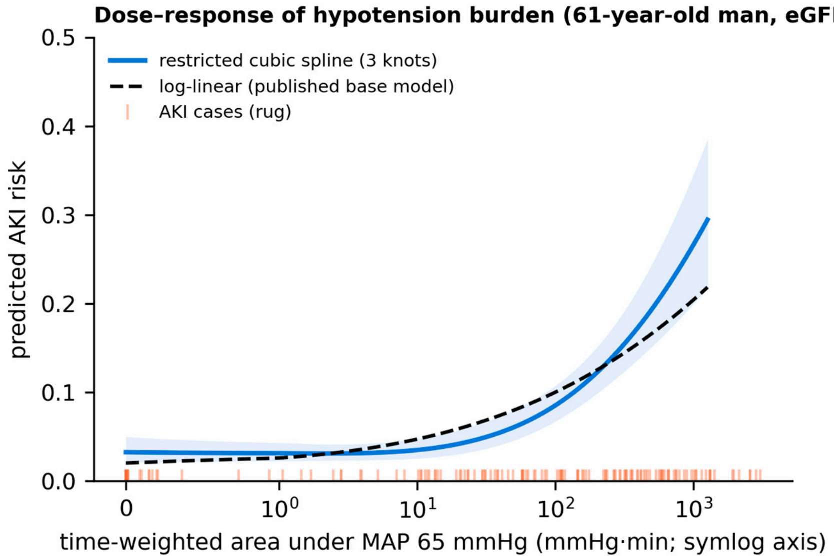

Predicted risk of AKI against the time-weighted area under MAP 65 mmHg for a 62-year-old man with an eGFR of 95 mL/min/1.73 m², under the log-linear base model (dashed) and under a restricted cubic spline with 3 knots (solid, with pointwise 95% confidence band); the rug marks the burden of patients with AKI.
